# The Taxonomic and Functional Diversity of Microbes at a Temperate Coastal Site: A ‘Multi-Omic’ Study of Seasonal and Diel Temporal Variation

**DOI:** 10.1371/journal.pone.0015545

**Published:** 2010-11-29

**Authors:** Jack A. Gilbert, Dawn Field, Paul Swift, Simon Thomas, Denise Cummings, Ben Temperton, Karen Weynberg, Susan Huse, Margaret Hughes, Ian Joint, Paul J. Somerfield, Martin Mühling

**Affiliations:** 1 Plymouth Marine Laboratory, Plymouth, United Kingdom; 2 Argonne National Laboratory, Argonne, Illinois, United States of America; 3 Department of Ecology and Evolution, University of Chicago, Chicago, Illinois, United States of America; 4 National Environment Research Council (NERC) Centre for Ecology and Hydrology, Wallingford, Oxfordshire, United Kingdom; 5 Josephine Bay Paul Center for Comparative Molecular Biology and Evolution, Marine Biological Laboratory, Woods Hole, Massachusetts, United States of America; 6 School of Biological Sciences, University of Liverpool, Liverpool, United Kingdom; 7 TU Bergakademie Freiberg, IÖZ - Interdisciplinary Centre for Ecology, Freiberg, Germany; Universidad Miguel Hernandez, Spain

## Abstract

How microbial communities change over time in response to the environment is poorly understood. Previously a six-year time series of 16S rRNA V6 data from the Western English Channel demonstrated robust seasonal structure within the bacterial community, with diversity negatively correlated with day-length. Here we determine whether metagenomes and metatranscriptomes follow similar patterns. We generated 16S rRNA datasets, metagenomes (1.2 GB) and metatranscriptomes (157 MB) for eight additional time points sampled in 2008, representing three seasons (Winter, Spring, Summer) and including day and night samples. This is the first microbial ‘multi-omic’ study to combine 16S rRNA amplicon sequencing with metagenomic and metatranscriptomic profiling. Five main conclusions can be drawn from analysis of these data: 1) Archaea follow the same seasonal patterns as Bacteria, but show lower relative diversity; 2) Higher 16S rRNA diversity also reflects a higher diversity of transcripts; 3) Diversity is highest in winter and at night; 4) Community-level changes in 16S-based diversity and metagenomic profiles are better explained by seasonal patterns (with samples closest in time being most similar), while metatranscriptomic profiles are better explained by diel patterns and shifts in particular categories (i.e., functional groups) of genes; 5) Changes in key genes occur among seasons and between day and night (i.e., photosynthesis); but these samples contain large numbers of orphan genes without known homologues and it is these unknown gene sets that appear to contribute most towards defining the differences observed between times. Despite the huge diversity of these microbial communities, there are clear signs of predictable patterns and detectable stability over time. Renewed and intensified efforts are required to reveal fundamental deterministic patterns in the most complex microbial communities. Further, the presence of a substantial proportion of orphan sequences underscores the need to determine the gene products of sequences with currently unknown function.

## Introduction

The diversity of bacteria, as revealed by 16S rRNA, is well-known to be extremely high [1]–[10]. Therefore it is expected that the functional (phenotypic) diversity of these organisms will also be vast. This is already evidenced by biogeographic studies [e.g. 11], [8] that highlight the huge store of microbial proteins present in marine communities. For example, Rusch and colleagues [11] found approximately 4.4 million unique genetic fragments in a study of 7.7 million sequences. However, to the best of our knowledge there have been no studies that have made direct comparisons of overall diversity at the 16S rRNA, metagenomic and metatranscriptomic levels over time.

Here we apply such a ‘multi-omic’ approach to begin to unravel relationships between genetic and functional diversity in a temperate coastal marine microbial community. Marine bacteria demonstrate seasonal patterns in diversity with, generally, higher diversity during the winter than the summer in pelagic ecosystems [12]–[14]. Numerous environmental factors have been suggested to influence this diversity (e.g. temperature and nutrients: [13], [10], [15]), yet our characterization of the long-term coastal ocean observatory site, L4, in the Western English Channel (http://www.westernchannelobservatory.org.uk/all_parameters.html) suggests that the robust seasonal pattern in species richness is most closely correlated to day length [14]. It is possible that an ability of organisms to respond to day length could explain the resilience of “metabolic circadian oscillators”, allowing organisms to respond to changing environmental conditions [16]. Recently, the transcriptional profiles from one pair of night and a day samples of bacterioplankton in the oligotrophic North Pacific Ocean were examined [17], showing that transcriptional activity was correlated to the diel cycle and the estimated diversity of the COG functional categories was higher at night. While previous genomic and metagenomic research has mainly focused on the diel rhythm in photosynthetic microorganisms [e.g. 18]–[20], we now extend this to the whole prokaryotic community, including the non-photosynthetic microorganisms, by comparing night- and day-time samples collected within the same 24 hr period.

Specifically, we test two hypotheses about the microbial community found at L4. Firstly, that bacterial and archaeal *functional potential* (genetic capacity) and *functional actuality* (transcriptional response) will track diversity (based on the 16S rRNA marker) and show similar seasonal patterns. Secondly, that within short time periods, metatranscriptomes will show more differences than 16S rRNA and metagenomic profiles, reflecting the relationship between expression of particular sets of genes and environmental variation, such as on a day/night cycle.

To test these hypotheses, datasets were generated from pelagic water samples taken from the L4 station during the day and night at 3 sampling time points in 2008: January, April and August, representing winter, spring and summer. Samples were collected once during day time and once during night in January and April, while in August four samples, taken at six hourly intervals, were collected over a 24-hour period. Bacterial and archaeal 16S rRNA V6 amplicon-pyrosequenced taxonomic profiling was used, to allow direct comparison of additional time points and the existing six year time series [10], [14] and to determine if Archaea show a similar seasonal trend to Bacteria. Shotgun sequencing of both DNA (metagenomes) and mRNA (metatranscriptomes) was employed to compare the taxonomic diversity with the *functional potential* (genetic capacity) and *functional actuality* (expressed genetic material) of the microbial community respectively, over time.

## Materials and Methods

### Water Sampling

Surface water (0–2 m) samples were collected from the L4 sampling station (50.2518° N, 4.2089° W), part of the Western Channel Observatory (WCO, http://www.westernchannelobservatory.org.uk), on January 28^th^, April 22^nd^, August 27^th^ and August 28^th^ 2008. In January a sample was taken at 1500 h at the L4 station. A minimal-impact surface buoy with a drogue at 7 m depth, was deployed to track the surface water mass on a Lagrangian sampling approach. At 1900 h, approximately 2 hours after total darkness, a second sample was taken at 50.2611° N: 4.2435° W. In April, an initial sample was taken at 1600 h at the L4 station and, following a Lagrangian drift, a second sample was taken at 2200 h (1.5 h after darkness) at 50.253°N: 4.1875°W. In August, four samples were taken over a 24 h period, again using a Lagrangian approach. Sampling began at 1600 h on the 27^th^ at L4, 2200 h (2 h after sunset) at 50.2545°N: 4.199°W, at 0400 h (2-hours before sunrise) on the 28^th^ at 50.2678°N: 4.1723°W, and at 1000 h at 50.2665°N: 4.1486°W.

For each sample, 20 L of seawater were collected from the surface (0–2 m) and pre-filtered through a Whatman GF/A filter (∼1.6 µm poresize). The filtrate was passed through a 0.22 µm Sterivex cartridge (Millipore) for a maximum of 30 minutes (approximately 10 L per Sterivex cartridge). Sterivex cartridges were pumped dry and then immediately snap-frozen in liquid nitrogen, transferred in liquid nitrogen back to the laboratory, barcoded [32], and stored at −80°C until nucleic acid extraction. Ambient water temperature, density, salinity, chlorophyll a, total organic nitrogen and carbon, nitrate, ammonia, silicate and phosphate concentration were also determined for each sampling occasion (**Table 1**). Methods used for determining these variables are available on the WCO website (http://www.westernchannelobservatory.org.uk/all_parameters.html).

**Table 1:** Environmental variables for each sampling occasion.

	28/01/2008	28/01/2008	22/04/2008	22/04/2008	26/08/2008	26/08/2008	27/08/2008	27/08/2008
Time	14:00	22:00	14:00	22:00	16:00	22:00	04:00	10:00
**Temperature (°C)**	10.1	10.05	9.7	9.6	15.9	15.8	15.7	15.8
**Density (kg m^−2^)**	1025.6	1026.3	1027.2	1027.1	1023.5	1024.3	1024.5	1024.4
**Salinity (PSU)**	33.3	34.2	35.12	35	32.1	33	33.3	33.2
**Chlorophyll a (µg/L)**	0.81	0.85	2.20	1.32	9.24	8.17	9.80	11.91
**Total Organic Nitrogen (µmol L^−1^)**	1.331	3.453	2.897	2.773	2.837	2.329	3.018	4.145
**Total Organic Carbon (µmol L^−1^)**	33.185	38.168	27.247	19.413	26.761	26.482	22.015	23.671
**NO_2_ + NO_3_ (µmol L^−1^)**	10.9	10.02	4.02	3.75	0.08	0.1	0.9	0.09
**Ammonia (µmol L^−1^)**	0	0	0.54	0.25	0.06	0.1	0.14	0.05
**SRP (µmol L^−1^)**	0.53	0.52	0.4	0.32	0.03	0.06	0.08	0.1
**Silicate (µmol L^−1^)**	6.01	5.75	2.6	2.7	0.12	0.22	0.33	0.15

### Nucleic Acid Extraction

DNA and RNA were isolated from each sample [25], [33], barcoded [32] and then stored at −80°C. DNA and mRNA-enriched cDNA were purified from the same pool using the techniques described in detail in Gilbert et al. [25]. DNA was used for metagenomic and 16S rRNA V6 amplicon pyrosequencing analysis and mRNA-enriched cDNA was used for metatranscriptomic pyrosequencing analysis. All DNA and cDNA were pyrosequenced using the GS-FLX Titanium platform. All data are available on the CAMERA website under ‘Western Channel Observatory Microbial Metagenomic Study (http://web.camera.calit2.net) and MG-RAST under 4443360-63; 4443365-68 and 4444077, 4445065-68, 4445070, 4445081, 4444083 (http://metagenomics.nmpdr.org/), as well as through the INSDC short reads archive under ERP000118 (http://www.ebi.ac.uk/ena/data/view/ERP0001180). All submissions conform to the “Minimum information standards” recommended by the Genomic Standards Consortium (**Table 1**; [34]; http://gensc.org/gc_wiki/index.php/MIENS).

### 16S rRNA V6 amplicon pyrosequencing

16S rRNA V6 amplicon pyrosequencing was carried out as described by Huber et al. [35] and Gilbert et al. [10]. For Bacteria, the primers (Table S1) were used in multiplex [35]. All 8 samples were run in the same GS-FLX 454 pyrosequencing reaction using multiplex identifiers (MIDs). These were January day (ACGAGTGCGT), January night (ACGCTCGACA), April Day (AGACGCACTC), April Night (AGCACTGTAG), August 4pm (ATCAGACACG), August 10pm (ATATCGCGAG), August 4am (CGTGTCTCTA), August 10am (CTCGCGTGTC). All amplicons were sequenced using the 454 Corporation's GS-FLX instrument at the NERC-funded Advanced Genomics Facility at the University of Liverpool (http://www.liv.ac.uk/agf/). Archaeal 16S rRNA amplicon pyrosequencing (for primer see Table S1) was performed at the International Census for Marine Microbes (ICoMM) Initiative laboratory at Woods Hole in September 2008 according to the method of Huber et al. [35], again using the GS-flx platform.

### 16S rRNA data processing

The 16S rDNA sequence reads were filtered [36] to remove all reads that did not have an exact match to the MID, the proximal primer or a near match to the distal primer, along with reads that contained Ns. Primer sequences were trimmed from both ends. Any reads less than 50 nt, or whose average quality score was less than 30 after trimming, were also removed. Taxonomic assignments were made to each trimmed high-quality sequence using GAST [37]. The V6 pyrosequencing reads were compared to a database of V6 region sequences excised from the SILVA reference database of full-length rDNA sequences of known taxonomy. The taxonomy of each sequence was assigned the consensus of the nearest reference V6 sequence(s).

Sequence-based clustering to create operational taxonomic units (OTUs) was based on the single-linkage preclustering (SLP) method introduced in Huse et al. [38]. To smooth some of the noise inherent in sequencing, a 2% single-linkage preclustering step was used to combine sequences likely to be variants from the same source amplicon. These clusters were then used as input to an average linkage clustering using MOTHUR [39] based on pairwise alignments using ESPRIT [40]. The final clusters were created using 3, 6, and 10% clustering thresholds.

Good's nonparametric coverage estimator was used to calculate the coverage obtained for the 16S rRNA V6 datasets using the formula C = 1−(*n*i/N)×100, where N =  total number of sequence reads analysed and *n*i  =  number of reads that occurred only once among the total number of reads tested [41], [42].

### Metagenomic and Metatranscriptomic profiling

Primary analyses were performed using the Metagenome Rapid Annotation using Subsystem Technology (MG-RAST) bioinformatics server [30]. Additional manipulations of the data used custom-written programming scripts (available at http://nebc.nerc.ac.uk/tools/scripts) and were processed on the Bio-Linux platform [43] unless otherwise indicated. For quality control the following sequences were removed from both metagenomes and metatranscriptomes (*sequence-filter.pl*): transcript fragments with >10% non-determined base pairs (Ns); fragments <75 bp in length, fragments with >60% of any single base; exact duplicates (which result from aberrant dual reads during sequence analysis). The removal of artificial duplicate sequences (i.e. multiple reads which start at exactly the same position in a metagenome from a complex ecosystem, e.g. [44]) from the pyrosequenced data was rejected on the basis that the majority of metatranscriptomic duplicates may very well be natural, and as such comparative analysis between metagenomic and metatranscriptomic data could not be performed if removal was applied to one and not the other [45].

All nucleic acid sequences were then compared against all three major ribosomal RNA databases (SILVA (http://www.arb-silva.de/); RDP II (http://rdp.cme.msu.edu/); Greengenes (http://greengenes.lbl.gov) using the bacterial and archaeal 5S, 16S and 23S, and the eukaryotic 18S and 25S sequence annotator function of MG-RAST (e-value <1×10^−5^; minimum length of alignment of 50 bp; minimum sequence nucleotide identity of 50%) and excluded if annotated as rRNA. All subsequent reads were considered to be valid DNA or valid putative mRNA derived sequences and were annotated against the SEED database using MG-RAST (e-value <1×10^−3^; minimum length of alignment of 50 bp; minimum sequence nucleotide identity of 50%; [30]) to produce an abundance matrix of functional genes and protein-derived predicted taxonomies across the DNA and mRNA samples. In addition all sequences were translated as previously [25], [11] producing predicted open reading frames (pORFs) in all six reading frames, using the rule that a pORF had to have more than 40 amino acids. All proteins from all datasets were then clustered together using CD-hit [46] at 95% amino acid identity over 80% of the length of the longest sequence in a cluster. Subsequently, the longest representative from each cluster was clustered at 60% amino acid identity over 80% of the length of the longest sequence to group these sequences by protein families [e.g. 25], [11]. The relative abundance of each sample in a cluster was used to create an abundance matrix using the output cluster files from the CD-HIT program, the files containing the original fasta sequences and headers for each sample (*abundanceMatrix-twoStep.pl)*. Subsequently, all protein clusters with ≤2 representative pORFs were removed from the pORF matrix (*MatrixParser.pv*). To allow direct comparison all samples were randomly re-sampled (*Daisychopper.pl*) to the same number of pORFs or sequences across the clusters or functional/taxonomic SEED annotations, to equalize the sequencing effort. The abundance of each pORF cluster and functional/taxonomic SEED annotation was then analysed using non-parametric multivariate analyses [47], [48]. Firstly, all data were transformed by square root; then a separate Bray-Curtis similarity matrix was calculated for the protein clusters and SEED annotations for both the metagenomes and metatranscriptomes. The matrices were then clustered using hierarchical agglomerative clustering with group- average linkage to produce a dendrogram representing the scaled similarity between samples. Similarity profiles analysis, SIMPROF [49] was used to test for multivariate structure. Nonmetric multidimensional scaling was used to ordinate similarities between samples. For the SEED annotations, similarities percentages breakdown, SIMPER [47] was used to determine which functional categories and SEED subsystems contributed most to differences between groups of samples (grouped by day/night and season). Good's coverage estimates (see above) were also calculated for metagenomic and metatranscriptomic pORFs as for 16S rRNA V6 sequences, where *n*i is the number of singleton pORFs.

## Results and Discussion

The bacterial community at the L4 site in the Western English Channel is seasonally structured, cycling through Winter, Summer, Autumn and Spring communities, and diversity shows a relationship with day-length [10], [14]. Here, we address whether metagenomic and metatranscriptomic profiles follow a similar pattern, extend our study of L4 to include time points taken at night and include Archaea in our analysis for the first time. Overall this study generated 5,720,488 sequences as summarized in **Tables 2**, **3**, **4** **and** **5**.

**Table 2:** Comparison of bacterial 16S rRNA V6 fragment datasets from day and night samples in January, April and August.

*Bacteria 16S tags*	January		April		August 27th		August 28th		Total
Time	15:00	19:30	16:00	22:00	16:00	22:00	04:00	10:00	
Original Sequences	5,945	5,294	7,064	4,970	6,956	6,914	10,413	9,782	57,338
Clustered Sequences	4,534	4,070	6,219	6,211	9,534	8,759	5,913	4,133	49,373
**Resampled sequencing effort (4070)**									
OTUs (4070)	331	558	177	199	189	174	192	189	999*
Number of singletons (4070) (%)	138 (42)	307 (55)	74 (42)	86 (43)	76 (40)	67 (39)	87 (45)	83 (44)	523* (52)
Good's Coverage (4070)	96.61	92.46	98.18	97.89	98.13	98.35	97.86	97.96	98.39

De-noising was performed using the SLP technique (Huse et al., 2010).

*Total number of OTUs when combining all datasets together: therefore, OTUs from combined dataset, not a sum of OTUs from individual datasets. Coverage  =  (1−(number of singletons/number of resampled sequences)) x 100.

**Table 3:** Comparison of archaeal 16S rRNA V6 fragment datasets from day and night samples in January and August.

*Archaea 16S rDNA*	January		April		August 27th		August 28th		Total
Time	15:00	19:30	16:00	22:00	16:00	22:00	04:00	10:00	
Original Sequences	57,030	25,075	nr	nr	17,822	27,882	nr	nr	127,809
Sequences following de-noising	54,058	23,900	nr	nr	16,771	26,317	nr	nr	121,046
**Resampled sequencing effort (16,771)**									
OTUs (16,771)	46	70	nr	nr	22	27	nr	nr	111*
Number of singletons (16,771) (%)	25 (54)	33 (47)	nr	nr	9 (41)	14 (52)	nr	nr	64* (58)
Good's Coverage (16,771)	99.85	99.80	nr	nr	99.95	99.92	nr	nr	99.90
**Resampled sequencing effort (4070)**									
OTUs (4070)	24	46	nr	nr	14	13	nr	nr	63*
Number of singletons (4070) (%)	7 (29)	20 (43)	nr	nr	2 (14)	5 (38)	nr	nr	31* (49)
Good's Coverage (4070)	99.83	99.51	nr	nr	99.95	99.88	nr	nr	99.81

De-noising was performed using the SLP technique (Huse et al., 2010).

*Total number of OTUs when combining all datasets together: therefore, OTUs from combined dataset, not a sum of OTUs from individual datasets. Coverage  =  (1−(number of singletons/number of resampled sequences))×100. Nr – not recorded.

**Table 4:** Comparison of metagenomic datasets from day and night samples in January, April and August.

*Metagenomes*	January		April		August 27th		August 28th		Total
Time	15:00	19:30	16:00	22:00	16:00	22:00	04:00	10:00	
No. Original DNA Sequences	616,793	784,823	637,801	493,003	620,759	524,953	500,117	326,475	4,504,724
Predicted ORFs (>40aa pORFs)	862,695	1,287,412	1,003,799	745,305	986,269	846,209	779,951	491,330	7,002,970
No. of pORF clusters (95%)	615,374	1,123,829	779,342	588,387	881,113	703,712	675,210	444,729	5,380,725** (7,002,970 seqs)
No. of pORF singletons (95%)	546,463	1,031,865	682,586	526,233	805,284	634,042	608,785	410,616	4,658,405** (4,658,405 seqs)
No. of pORF ‘families’ (60%)	423,674	1,031,904	678,547	528,213	801,760	637,542	620,403	419,461	4,418,324** (5,380,725 seqs)
No. of pORF singletons (60%)	352,938	962,073	609,351	486,712	740,032	589,839	577,027	398,202	3,822,888** (3,822,888 seqs)
**Resampled pORFs (66529)**									
No. of pORF clusters (95%) (66529)	56337	64446	61187	59904	65601	63032	64729	65075	488903** (532232 seqs)
No. of pORF singletons (95%) (66529)	52891	63378	58691	57779	64818	61068	63359	63945	468,449
Good's Coverage (66529)	20.50	4.74	11.78	13.15	2.57	8.21	4.76	3.88	4.18
No. DNA seqs with functional annotation	122,936	291,953	258,658	164,249	283,761	196,369	196,972	126,392	1,641,290
No. DNA seqs without functional annotation (%)	493,857 (80)	492,870 (63)	379,143 (59)	328,754 (67)	336,998 (54)	328,584 (63)	303,145 (61)	200,083 (61)	2,863,434 (64)
No. DNA seqs with taxonomic annotation	190,326	417,920	349,888	241,541	379,911	288,356	304,003	186,421	2,358,366
**Resampled sequencing effort (186,421)**									
Number of archaeal sequences (186,421)	19,055	15,150	777	561	1,370	1,093	1,585	1,244	40,835
Number of bacterial sequences (186,421)	161,899	146,911	182,850	180,674	182,717	176,825	180,725	182,332	1,394,933

**Number of protein clusters found when combining all datasets together: therefore, protein clusters from combined dataset, not a sum of clusters from individual datasets. Coverage  =  (1−(number of singletons/number of resampled sequences)) x 100.

**Table 5:** Comparison of metatranscriptomic datasets from day and night samples in January, April and August.

Metatranscriptomes	January		April		August 27th		August 28th		Total
Time	15:00	19:30	16:00	22:00	16:00	22:00	04:00	10:00	
No. Original cDNA Sequences	139,880	130,826	124,925	147,492	139,375	193,254	154,865	nr	1,030,617
No. of sequences following filtering***	94,024	106,864	84,916	109,577	87,799	118,360	111,568	nr	713,108
No. mRNA following removal of rRNA	61,831	96,026	41,378	53,413	33,149	51,829	55,006	nr	392,632
Predicted ORFs (>40aa pORFs)	143,169	211,374	81,642	107,699	77,985	66,529	159,909	nr	848,307
No. of pORF clusters (95%)	98,871	78,278	35,648	51,088	28,167	24,136	68,080	nr	350335** (848,370 seqs)
No. of pORF singletons (95%)	82,464	54,870	25,925	38,960	19,600	17,177	50,246	nr	262,767** (262,767 seqs)
No. of pORF ‘families’ (60%)	84,598	45,049	19,131	37,628	15,146	12,735	41,480	nr	230,505** (350,335 seqs)
No. of pORF singletons (60%)	76,655	30,720	13,869	30,919	9,857	9,134	32,662	nr	187,083** (187083 seqs)
**Resampled pORFs (66529)**									
No. of pORF clusters (95%) (66529)	31026	50354	30334	34217	24848	24136	33191	nr	205368** (465703 seqs)
No. of pORF singletons (95%) (66529)	23038	43687	22394	26840	17373	17177	25636	nr	157658
Good's Coverage (66529)	65.37	34.33	66.34	59.66	73.89	74.18	61.47	**nr**	66.15
No. mRNA seqs with functional annotation	11,513	31,990	8,845	16,315	11,720	5,907	15,384	nr	101,674
No. mRNA seqs without functional annotation (%)	50,318 (81)	64,036 (67)	32,533 (79)	37,098 (69)	21,429 (65)	45,922 (89)	39,622 (72)	nr	290,958 (74)
No. mRNA seqs with taxonomic annotation	29,521	30,778	20,899	26,398	15,456	29,605	38,304	nr	190,961
**Resampled sequencing effort (15,456)**									
Number of archaeal sequences (15,456)	625	49	1	16	4	4	11	nr	710
Number of bacterial sequences (15,456)	13,633	11,926	13,702	8,449	14,469	15,071	14,803	nr	92,053

**Number of protein clusters found when combining all datasets together: therefore, protein clusters from combined dataset, not a sum of clusters from individual datasets.

***filtering out any sequence with >10% Ns; <75 bp; >60% any single base; absolute duplicates. Coverage  =  (1−(number of singletons/number of resampled sequences)) x 100. Nr – not recorded.

### Bacterial and archaeal diversity show seasonal patterns, and detectable archaeal richness is lower

A total of 49,373 bacterial 16S rRNA V6 sequences were produced from eight samples, varying from 4,070 to 9,534 sequences per sample (**Table 2**). Following random re-sampling, to equalize sample size to that of the smallest sample (4070 sequences) and enable comparison based on equal sequencing effort [10], and de-noising (i.e. the removal of spurious sequences based on the SLP technique), the bacterial community contained 999 distinct OTUs (please refer to methods for the level of demarcation for the OTUs). Three dominant phyla account for 97% of the total bacterial OTUs, Proteobacteria (86%), Bacteroidetes (9%) and Cyanobacteria (2%).

Archaeal diversity, previously unstudied at the L4 site, was examined for a subset of samples, the two paired sets of samples taken January 28^th^ and August 27^th^ 2008 (i.e. four of the eight samples in this study). A total of 121,046 16S rRNA V6 sequences were generated, ranging from 16,771 to 54,058 per sample (**Table 3**). In terms of detected OTUs, archaeal diversity was much lower than bacterial diversity. Both bacterial and archaeal diversity (richness, S) peaked in winter **(** **Tables 2**, **3**, **4**, **5**, **6**), confirming previous findings [14]. After random-resampling of the archaeal data to adjust the size of all four samples to the smallest (16,771 sequences), the combined observed OTUs (S) for the four samples was only 111. The archaeal community was dominated by the Euryarchaeota (81%). Winter samples contained a larger number of OTUs (day + night average of 58) with the night time sample from January containing the highest observed number of OTUs (70). August showed 20 OTUs in the day-time and 27 OTUs at night. Following resampling of the archaeal dataset to the smallest bacterial dataset (4080 sequences), total archaeal diversity (richness) was only 6% that of bacterial diversity (63 versus 999 OTUs), with a maximum of 8% of the bacterial OTU richness in winter (January) (**Table 3**).

**Table 6:** Bacterial and archaeal diversity (S), species richness (d) and evenness (1-λ') for each sample.

Sample	Bacteria			Archaea		
	S	d	1-λ'	S	d	1-λ'
January day	331	39.7	0.8901	24	2.767	0.8271
**January night**	**558**	**67.02**	**0.9577**	**46**	**5.414**	**0.798**
April day	177	21.18	0.8289			
**April night**	**199**	**23.82**	**0.8745**			
August day 4pm	189	22.62	0.9355	14	1.564	0.6347
**August night 10pm**	**174**	**20.81**	**0.9341**	**13**	**1.444**	**0.628**
**August night 4am**	**192**	**22.98**	**0.9265**			
August day 10am	189	22.62	0.9247			

Diversity (S) is a measure of the number of unique OTUs derived from the 16S rRNA V6 pyrosequences. All datasets were randomly resampled to 4070 sequences, the size of the smallest archaeal dataset, to allow direct comparison of these bacterial and archaeal datasets. January Night has the highest evenness.

**Figure 1:**
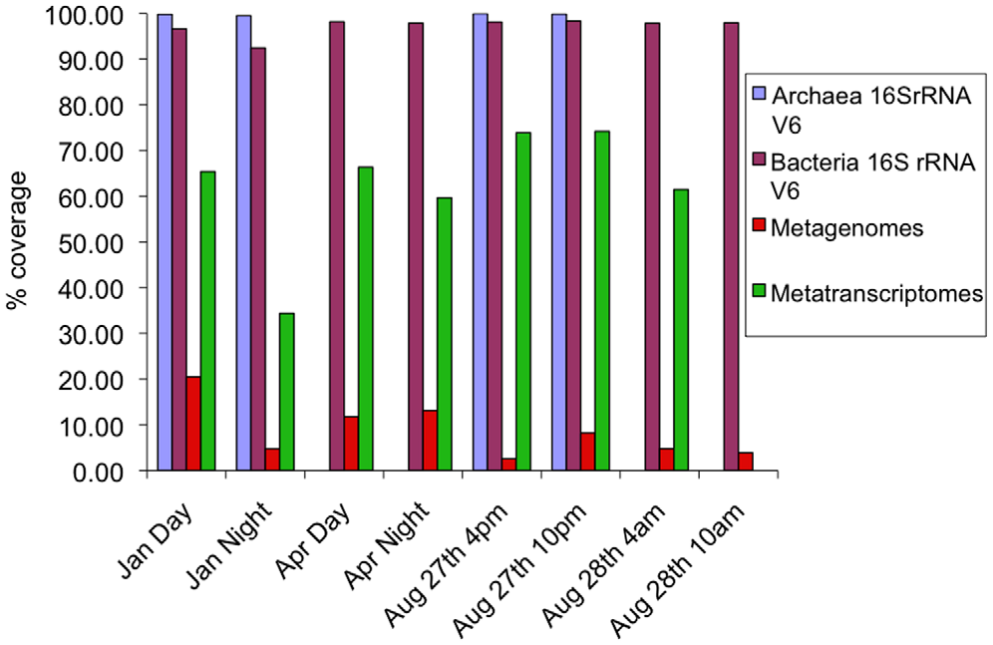
Good's estimate of coverage for bacterial and archaeal 16S rRNA V6 sequences resampled to 4,070 reads per sample (the smallest dataset), and for metagenomic and metatranscriptomic samples resampled to 66,529 (the smallest dataset). Values in **Table 2**.

Coverage of both bacterial and archaeal dominant taxa (defined as non-singleton OTUs) was high, but archaeal communities were better sampled. Using a uniform sample size of 4070 sequences (the smallest 16S rRNA dataset in the study), estimated coverage for bacteria ranged from a minimum of 92.5% of OTUs (night in January) to a maximum of 98.4% (10pm in August) (**Figure 1**; **Table 2**). For Archaea, all samples reached almost complete coverage (between 99.5% and 99.9% coverage) (**Figure 1**; **Table 3**). Additional sequencing of Archaeal 16S rRNA V6 fragments (when re-set to 16,771, the smallest Archaea dataset) beyond these 4070 sequences added only a few very rare taxa; for example, it did not increase coverage for the sample from August-4pm (already 99.95% coverage) while a maximum of 0.3% was added for the January-night sample (**Figure 1**; **Table 3**). This confirms previous studies that suggested that the majority of the abundant, dominant microbes can be captured with as few as 2000 sequences [21].

These results show that bacterial and archaeal diversity was highest in winter and that the majority of the abundant taxa were sampled in this study with only the very rare taxa remaining undetected. The largest numbers of singletons were found in winter (compared to spring and summer) and in night-time (compared to day-time) samples (**Table 2** **–** **3**). The lowest estimates of coverage were for winter and night-time samples. Of course, these estimates of diversity may be biased by the coverage of the primers and amplification conditions, and key lineages could possibly remain undetected [22].

**Figure 2:**
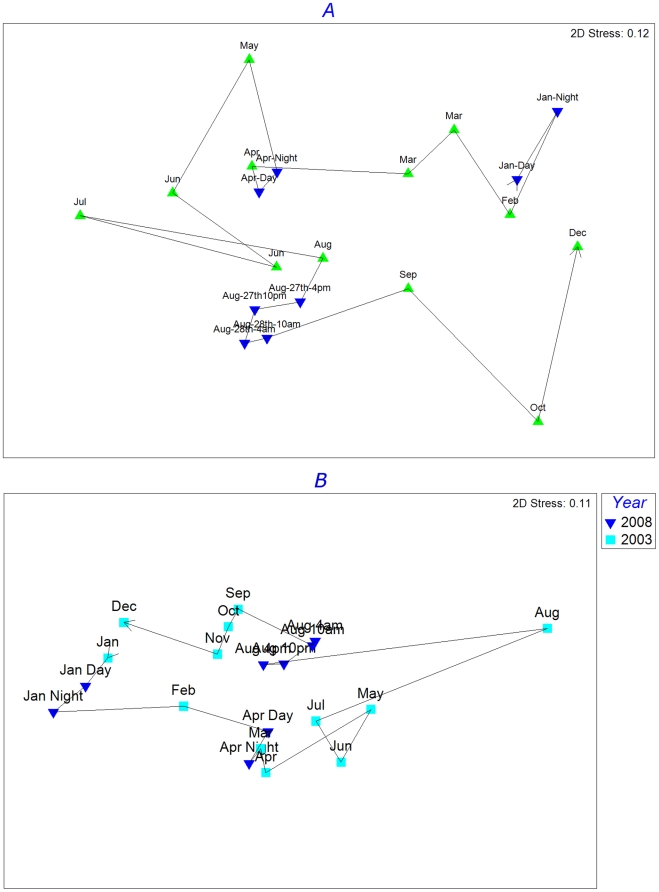
Non-parametric multidimensional scaling plot of similarities among 12 monthly sampling points from (A) 2008 and (B) 2003 (Gilbert et al., 2010) combined with the eight time points from the current study, based on square root transformed 16S rRNA V6-derived bacterial abundances and the Bray-Curtis similarity measure. Blue: samples from the current study; green: samples from the previous study. The line links adjacent samples in time from January to December. Day and night samples are shown in chronological order with day first and night second.

We also examined the relative similarities of OTUs among time points. First, we made a direct comparison between the eight samples analysed in this study and the 12 monthly samples from L4 which were also collected in 2008, included in Gilbert et al. [14]. Nonmetric multi dimensional scaling (NMDS) analysis of the combined 20 samples showed the strong seasonal structuring of the community at L4 (**Fig. 2**). Day and night samples within the same 24 hour period were similar, although diel differences were greatest in winter (**Fig. 2**). Strikingly, intra-annual patterns were so strong that these eight new data point could be equally well be placed into any year of data collected from L4 – an example from 2003 is shown in **Fig. 2B**.

**Figure 3:**
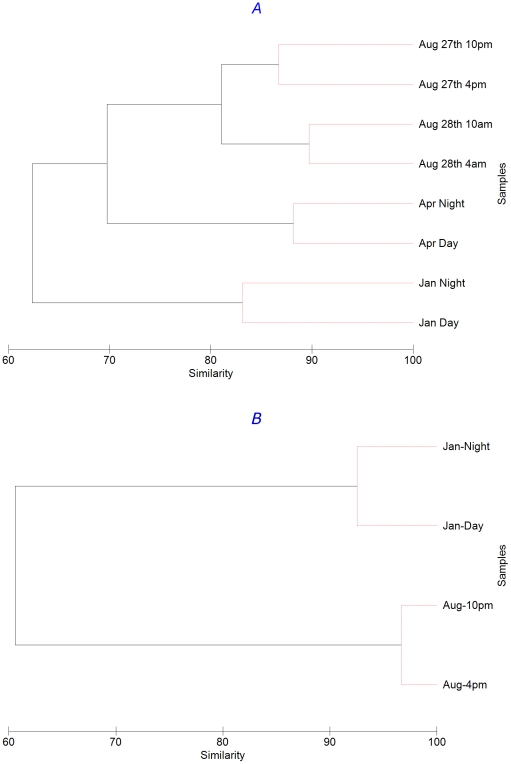
Bacterial (A) and archaeal (B) community comparison for each time point examined using group-average clustering of data from Bray-Curtis similarity matrices. All samples were randomly-resampled to 4070 sequences; abundance data were transformed by square root. SIMPROF testing has been applied to branching structure: red lines indicate branches in which re-arrangement indicates no significant difference between communities. Note, the test cannot discriminate between pairs of samples.

Further, hierarchical cluster analysis confirms that samples taken on the same date (day and night) were more similar than those taken from different seasons. For example, for Archaea, January and August daytime samples were only 52% similar, while similarities between day and night samples in winter and summer were 82% and 94%, respectively (**Fig. 3B**). Likewise, for bacteria January and August, samples were only 36% similar but day and night samples were 60% and 70% similar, respectively (**Fig. 3A**). Interestingly, for both Archaea and Bacteria divergence between day and night samples was greater in the winter samples compared to the summer samples (a shift from 82% to 94% for Archaea and 60% to 70% for Bacteria from January to August, respectively).

Despite smaller diel changes, compared to inter-seasonal changes, these data show that dramatic shifts in community composition could occur on time scales as small as a matter of hours. For example, despite being only a day apart, bacterial communities on August 27^th^ and 28^th^ were only 64% similar and were significantly different according to the SIMPROF analysis (**Fig. 3A**). SIMPER analysis suggests that 10% of the difference is due to an 80 fold increase in the abundance of a *Vibrio* organism, and a 68 fold increase in an *Alteromonas* organism from the 27^th^ to the 28^th^ August. However, even following removal of these organisms the communities are still significantly different, suggesting that the differences are not due to a bloom of one or two organisms, but rather a fundamental shift in community composition.

To explore the relative roles of dominant and rare taxa further, we tested whether the seasonal differences would remain if rare taxa were removed. Removing all OTUs with an abundance of less than 100 sequences (an arbitrary cut-off selected after inspection of the data) still yielded a statistically significant difference between seasons for both Bacteria and Archaea (ANOSIM R = 1.0, *p<*0.01). Observed differences between day and night were considerably reduced following the removal of rare taxa (e.g. the similarity between day and night bacterial communities increased for January from ∼59% to ∼83%, for April from 69% to 88%, and for August from on average 72% to 88%) (**Fig. S1A&B**). Second, we generated dendrograms from presence/absence transformation of the data to up-weight the role of the rarer taxa in the analysis. This led to a considerable decrease in similarity between all time points for both Bacteria and Archaea (**Fig. S1C&D**). In Archaea it also led to a near-complete breakdown in similarity between day and night, with January day showing greater similarity to the August (day as well as night) communities (**Fig. S1D**).

**Figure 4:**
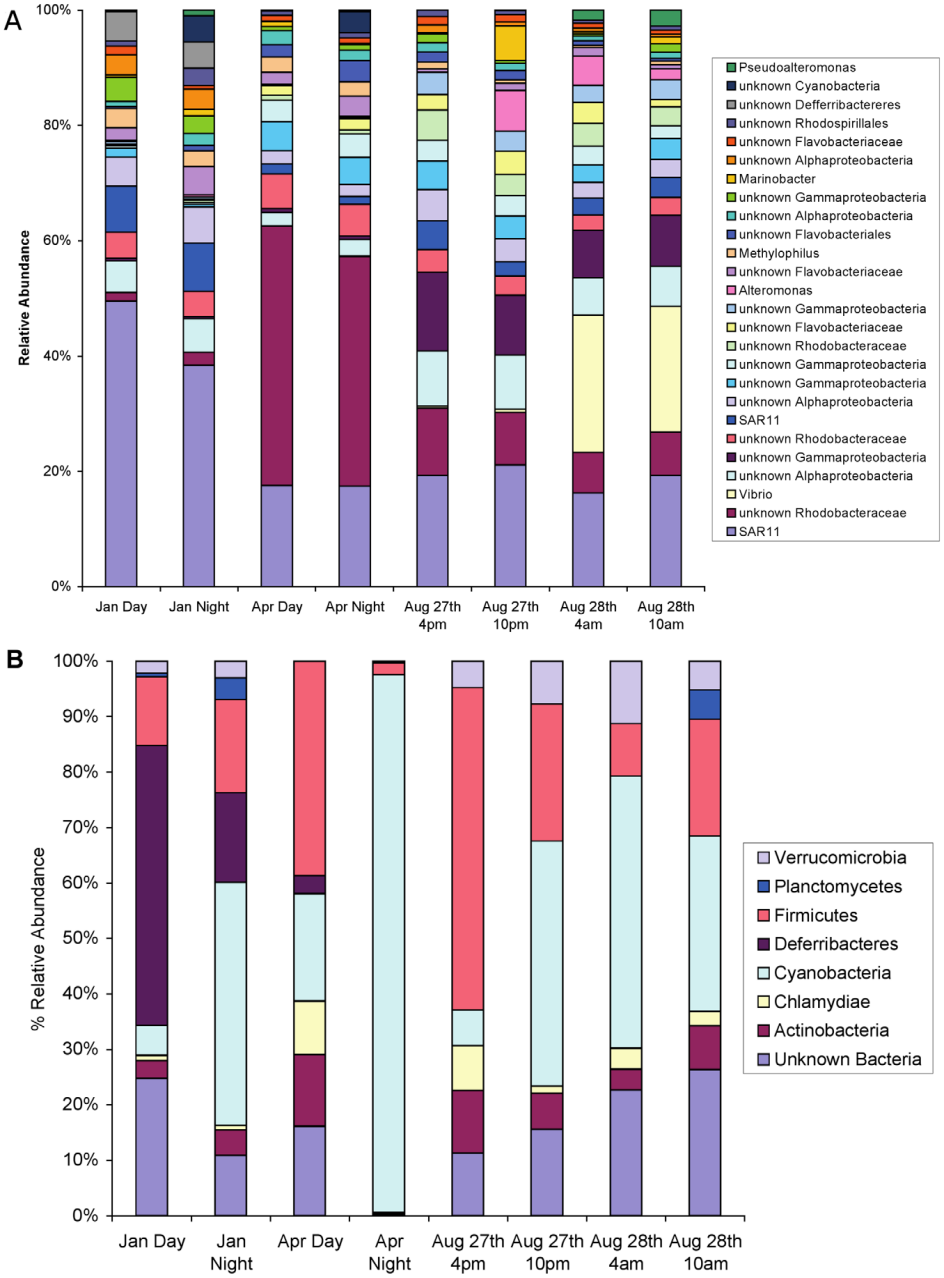
Percentage relative abundance of bacterial 16S rDNA V6 tags annotated to (A) all OTUs with an abundance of greater than 200 sequences and (B) all phyla with >10 sequences (sum of all 8 datasets) following removal of the *Proteobacteria* and *Bacteroidetes*. All analyses were performed following random resampling to 4070 16S rRNA V6 sequences per dataset.

**Figure 5:**
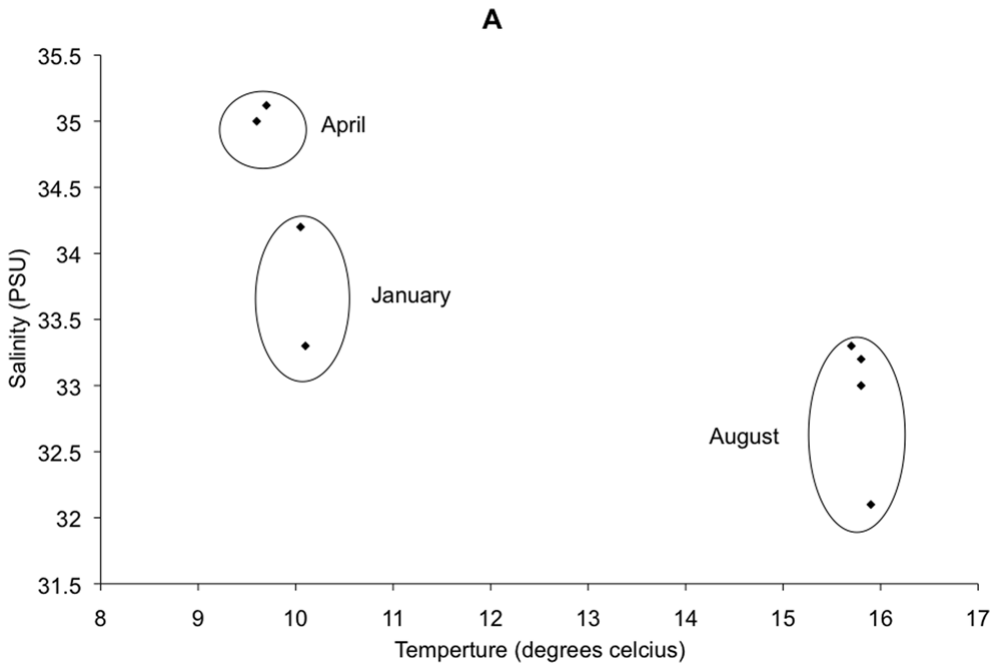
Relationship between salinity and temperature for the 8 sampling points, demonstrating that each month sampled represents different conditions. The outlier in August is the 4pm-27^th^ August sample.

Thus, both dominant and rare taxa show significant shifts between day and night samples. Three key examples support this observation. Firstly, in January, the overall most dominant organism (the most abundant OTU), which belongs to the SAR11 clade and comprised 25% of the community, showed a 40% reduction (from 1285 to 743 sequences) from day to night (**Fig. 4A**). Secondly, in April, the night sample had a 17% reduction (1528 to 1268 sequences) in the second overall most abundant OTU, an unknown *Rhodobacteraceae* organism (**Fig. 4A**). Thirdly, the 3^rd^ most abundant phylum, the Cyanobacteria, had a 15 fold day-to-night increase in January (17 to 258 sequences) and a 59 fold increase (6 to 350 sequences) in April (**Fig. 4B**), perhaps as a result of nocturnal cell division due to the well-known circadian rhythm of most marine Cyanobacteria [e.g. 23], [24], [20]. It is also possible that these differences result from the small changes in the salinity and temperature of the water body (Figure 5).

### Functional richness in metagenomic and metatranscriptomic samples is highest in winter and at night

We analyzed ∼4.5 million combined microbial metagenomic reads, comprising ∼1.9 billion base pairs (bp) with an average read length of ∼350 bp across the eight samples, ranging from 326,475 to 784,823 sequences per sample (**Table 4**). Seven metatranscriptomic datasets were also produced (the sample taken on August 28^th^ at 10am was lost in transit) totaling ∼1 million sequences. Following ‘clean-up’ (see Materials and Methods), a total of 392,632 putative mRNA-derived sequences remained, totaling 159 million bp with an average of 272 bp per sequence. The sequencing effort for the metatranscriptomic analyses varied from 33,149 to 96,026 sequences per sample (**Table 5**).

For metagenomes and metatranscriptomes we used the number of predicted protein clusters to assess and compare gene/transcript richness (diversity). All predicted open reading frames (pORFs) were identified from the metagenomic and metatranscriptomic datasets, after random resampling to 66,529 sequences (smallest metatranscriptomic pORF dataset; this is larger than the smallest mRNA sequencing effort, because one sequences can yield more than one open reading frame) and then clustered into families at 95% amino acid identity (as per [25]; **Table 4** **–** **5**; **Fig. S2**). For metatranscriptomes, there was a clear seasonal trend with the winter samples showing more pORF clusters compared to the spring and summer samples (**Table 5**; **Fig. S2).** This pattern in transcript richness was strongly correlated to the corresponding 16S rRNA V6 richness (R = 0.87 for the 7 samples analysed for both diversity and transcriptomes). The highest average (day and night) number of unique pORFs among metatranscriptomes was in winter (41,856) with lower numbers in spring (34,057) and summer (29,880). Transcript diversity was higher at night compared to daytime samples within each season (**Table 5**; **Fig. S2**).

In contrast, the number of average (day and night) observed metagenomic pORF clusters varied little across seasons (spring  = 64,161, summer  = 62,959 and winter  = 60,743). This 3.5% average change in the abundance of metagenomic pORFs across seasons (day/night averaging; Jan–Apr  = 5%; Apr–Aug  = 2%; Jan–Aug  = 3.5) is low in comparison to the 20% average change in metatranscriptomes (day and night averaging; Jan–Apr  = 19%; Apr–Aug  = 12%; Jan–Aug; 29%). Although the observed lack of difference among metagenomes could be real it must be seen in the context of the low coverage obtained (**Fig. 1**). Good's estimator of coverage for these datasets, resampled to the size of the smallest dataset (66,529 – Aug 27th 10pm metatranscriptome), showed that compared to high coverage of 16S rRNA V6 sequences (see above), metatranscriptomes had an average coverage of only 62% and metagenomes only 8%. As expected, during January and April, metatranscriptomic coverage was greatest during the day when diversity was lowest (**Fig. 1**). However, the metagenomic samples from August had the lowest coverage, and the metatranscriptomes from August had the highest coverage, which correlated with an increase in the abundance of bacterial cells during the summer at L4. Typically, bacterial and archaeal cell numbers were less than 500,000 cells per millilitre in winter, between 500,000–800,000 in spring, but could reach >2 million during June–August [26]. With a four fold increase in cellular abundance, mitigated by an increase in dominance (**Table 6**), a decrease in metagenomic coverage is expected; conversely the sustained metatranscriptomic coverage is likely to be a direct result of the increased dominance of specific taxa, and hence specific transcripts.

### Metagenomic profiles reflect seasonal trends associated with the 16S rRNA V6 analysis, but metatranscriptomes are more obviously structured by day/night changes

**Figure 6:**
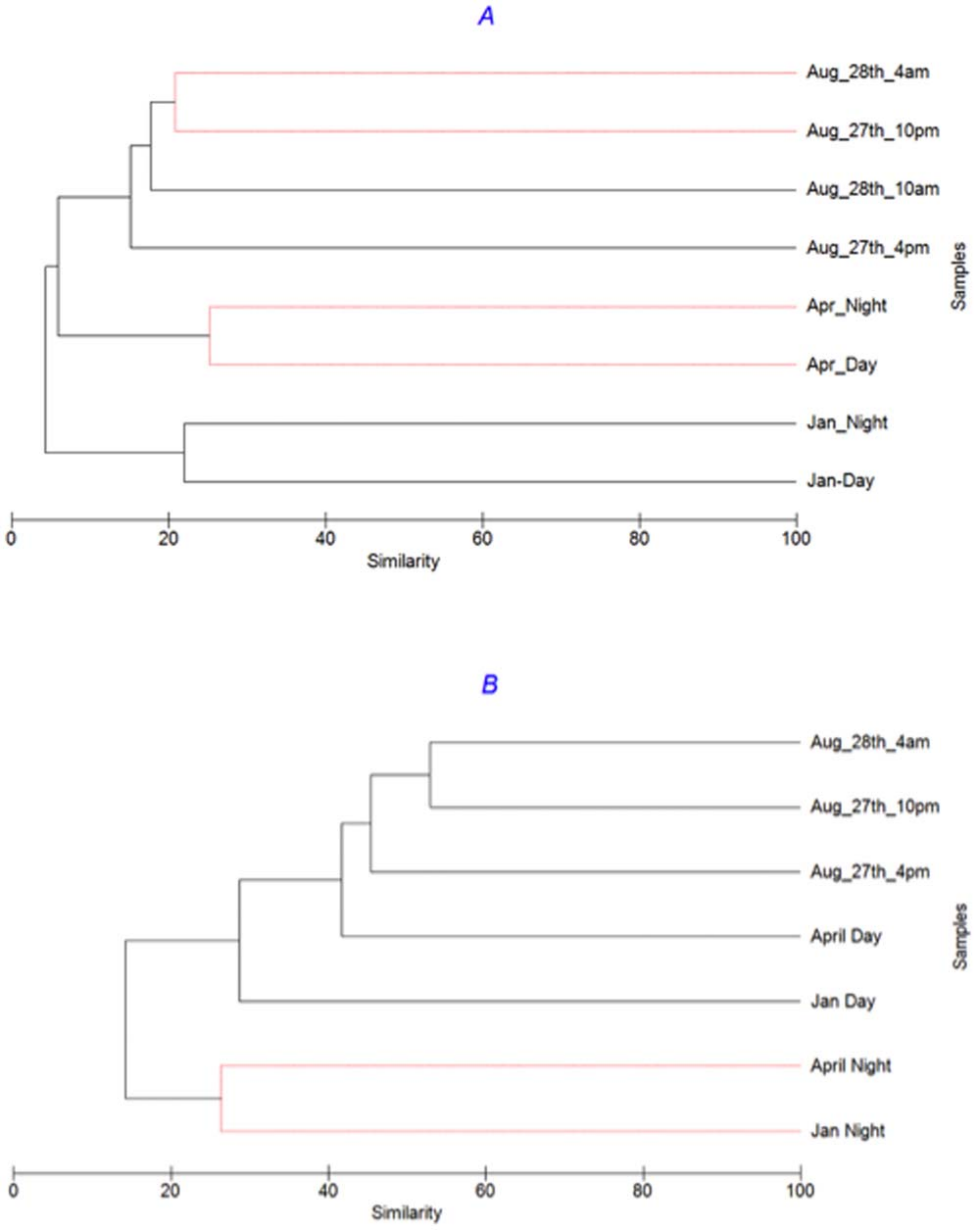
(A) metagenomic predictions of protein families (60% clustering), (B) metatranscriptomic predictions of protein families (60% clustering). All comparisons based on random resampling of metagenomic and metatranscriptomic datasets to 66,529 sequences (smallest dataset). SIMPROF testing has been applied to branching structure: red lines indicate branches in which re-arrangement indicates no significant difference between communities.

Dendrograms derived from similarities among pORF groups based on sequence similarity (60% amino acid identity over 80% length) (**Fig. 6A&B**) show that metagenomic pORF profiles were very dissimilar, with similarities of <2% in all instances. Despite this high dissimilarity between samples, the 8 samples grouped into clusters by season; that is day and night samples from the same sampling day showed the greatest similarity (**Fig. 6A**), agreeing with the results from the 16S rRNA V6 comparison (**Fig. 3**). Conversely, the same analysis with the metatranscriptomic data did not reveal any clear pattern (**Fig. 6B**). For example, the night time pORF transcriptomic profile from January and April were more similar to each other than the corresponding day-time samples from those two months which, however, did not group together. Some seasonal grouping of samples was still evident, as the three August samples were most similar to each other. However, SimProf analysis demonstrated that the August 27^th^ 2200 h and 28^th^ 0400 h night-time samples were statistically different to August 27^th^ 1600 h day time sample (**Fig. 6B**). This shows that diel differences in metatranscriptomic profiles were of the same order of magnitude, or greater, than seasonal differences.

### Specific types of genes, most notably photosynthetic genes, show seasonal and diel differences in abundance

**Figure 7:**
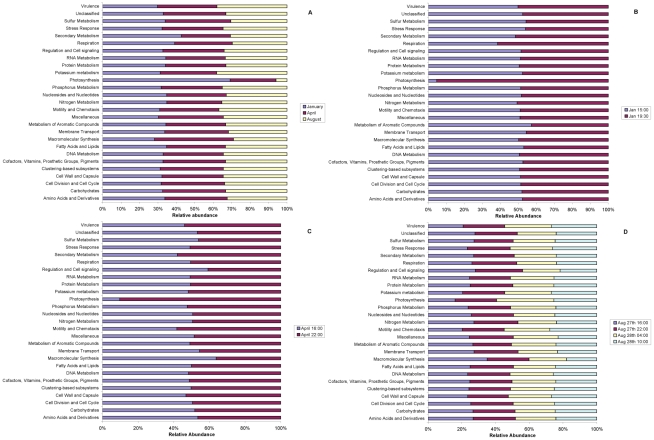
The average relative abundance metagenomic reads annotated to each hierarchy I subsystem from the SEED database compared between (A) each season and day and night for (B) January, (C) April and (D) August. Each dataset was randomly re-sampled prior to analysis.

**Figure 8:**
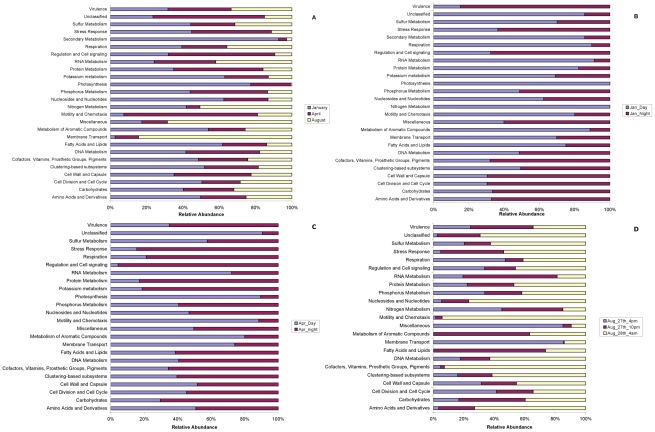
The average relative abundance of each metatranscriptomic fragments annotated to hierarchy I subsystem from the SEED database compared between (A) each season and day and night for (B) January, (C) April and (D) August. Each dataset was randomly re-sampled prior to analysis to the smallest metatranscriptome.

Plots of the relative abundances of all SEED hierarchical functional categories in seasonal and day and night samples are shown for metagenomes (**Fig 7**) and metatranscriptomes (**Fig 8**). Overall, as expected, metagenomes were more similar across all samples than metatranscriptomes.

Interestingly, photosynthesis genes showed changes over time that reflects greater photosynthetic potential in the winter, and in the day-time relative to night-time. SIMPER analysis showed that shifts in photosynthesis genes contributed most to differences in community composition among seasons (**Fig. 7A**; **Table S2**; e.g. photosystem I, II and cytochrome B6-F **Table S4**). In fact photosynthetic genes were ten times more abundant in winter than in summer (**Fig. 7A**). As the metagenomes were produced from DNA isolated from organisms smaller than 1.6 µm, the observed changes probably reflect changes among picophytoplankton, so this corroborates a previous observation that winter conditions promote the development of pico- and micro-phytoplankton over macro-phytoplankton when compared to summer conditions [27]. Photosynthetic genes were also considerably more abundant at night in January and April (**Fig. 7B&C**). Interestingly, gene fragments annotated to proteorhodopsin had a stable abundance throughout the year, approximately 0.07% of the annotated functional profile from each sample (data not shown), demonstrating no seasonal or diel fluctuation. The metatranscriptomic photosynthetic profiles followed similar patterns to the metagenomes, with genes for photosynthesis being most abundant in January compared to April, and virtually absent in August (**Fig. 8A**). Photosynthetic transcripts were most abundant in the day in all months; also they were most abundant during the winter, compared to the spring, compared to the summer following the abundance of the genes.

Other seasonal differences in metagenomic profiles were highlighted by SIMPER analysis, including considerably higher winter abundance (compared to spring or summer) of archaeal genes associated with lipid synthesis, thermosome chaperonins, RNA polymerase, small subunit ribosomal proteins, DNA replication and rRNA modification (**Table S4**). Archaea constituted a greater proportion of the genetic pool in winter (January) compared with April and August (**Table 6**). Archaea have been previously found to be more abundant and diverse during the winter in Arctic communities [28]. Diel differences were apparent among genes involved in respiratory metabolism, which were more abundant at night (**Fig. 7B**). In contrast to those observed changes, there was little difference in the metagenomic profile between day and night in April (**Fig. 7C**) and August (**Fig. 7D**), suggesting a very stable functional potential. SIMPER analysis (**Tables S5, S5, S7**) suggested that during August genes involved in virulence contributed ∼11% to the differences between day and night (**Table S3**), and the most significant contributors within this group of virulence related genes were proteins involved in iron uptake and metabolism, as well as cadmium, zinc and cobalt resistance (**Table S7**).

Other seasonal differences in the metatranscriptomic seasonal profiles included a greater relative abundance of transcripts related to membrane transport, especially amino-acid transport, in summer when nutrients and DOM are least abundant (**Fig. 8A**; **Table 1**; **Fig S3**). SIMPER analysis suggested that these differences contributed 13% of the dissimilarity between January and August samples (**Table S8**). The diel metatranscriptional profiles for January demonstrated considerable difference in functions (in addition to photosynthesis) between day and night; for example, transcripts relating to nitrogen cycling were found mostly during the day and were primarily associated with ammonification (**Fig. 8B**). Cell wall and capsule, and cell division and cycle were upregulated at night, suggesting a nocturnal increase in cell division, potentially associated with the Cyanobacteria. SIMPER analysis also indicated that the daytime upregulation of RNA and protein metabolism and the night time upregulation of virulence transcripts accounted for ∼34% of the dissimilarity between day and night (**Table S9**). Similarly, April samples (**Fig 8C**) showed a considerable upregulation in RNA metabolism during the day, primarily resulting from an increase in group I intron and RNA polymerase transcripts (**Fig 8C**). SIMPER analysis indicates that 30% of the dissimilarity was contributed by changes in RNA and protein metabolism and cellular regulation and signaling (**Table S9**); interestingly, protein metabolism was driven by a night-time upregulation in universal GTPases suggesting a rapid shift in ribosomal activity at night. In August, transcripts with homology to membrane transport (as discussed above) were upregulated during the day, while transcripts associated with motility and chemotaxis, and the synthesis of cofactors, vitamins, prosthetic groups and pigments were considerably upregulated at night, suggesting that nocturnal motility and cellular activity (nucleotide and amino acid synthesis) were also upregulated; **Fig 8D**). Membrane transport, RNA and carbohydrate metabolism (primarily driven by catabolism of ribose and deoxyribose sugars) contributed ∼39% to these differences (**Table S9**).

### The majority of differences between metagenomic and metatranscriptomic samples are due to orphaned genes and transcripts

As in most metagenomic and metatranscriptomic projects involving diverse communities [e.g. 29], [25], [17], only a small fraction of the predicted proteins identified in this study could be assigned an annotation using SEED subsystems [30]. SEED subsystems are largely expected to best cover house-keeping genes and the best studied gene families [31]. For this study, coverage of pORFs by SEED annotation ranged from 20–46% of each metagenomic dataset and 11–35% of the metatranscriptomic datasets (**Table 1**). There was no overall relationship between the observed coverage of each sample and the ability to annotate functions for each sequence (**Fig. S4**). However, the percentage of annotated transcripts was highest in the night compared to day samples from January and April (although this was not observed in August), which inversely correlated with a reduction in metatranscriptomic coverage (R = −0.79, p<0.05; **Fig. S4**).

**Figure 9:**
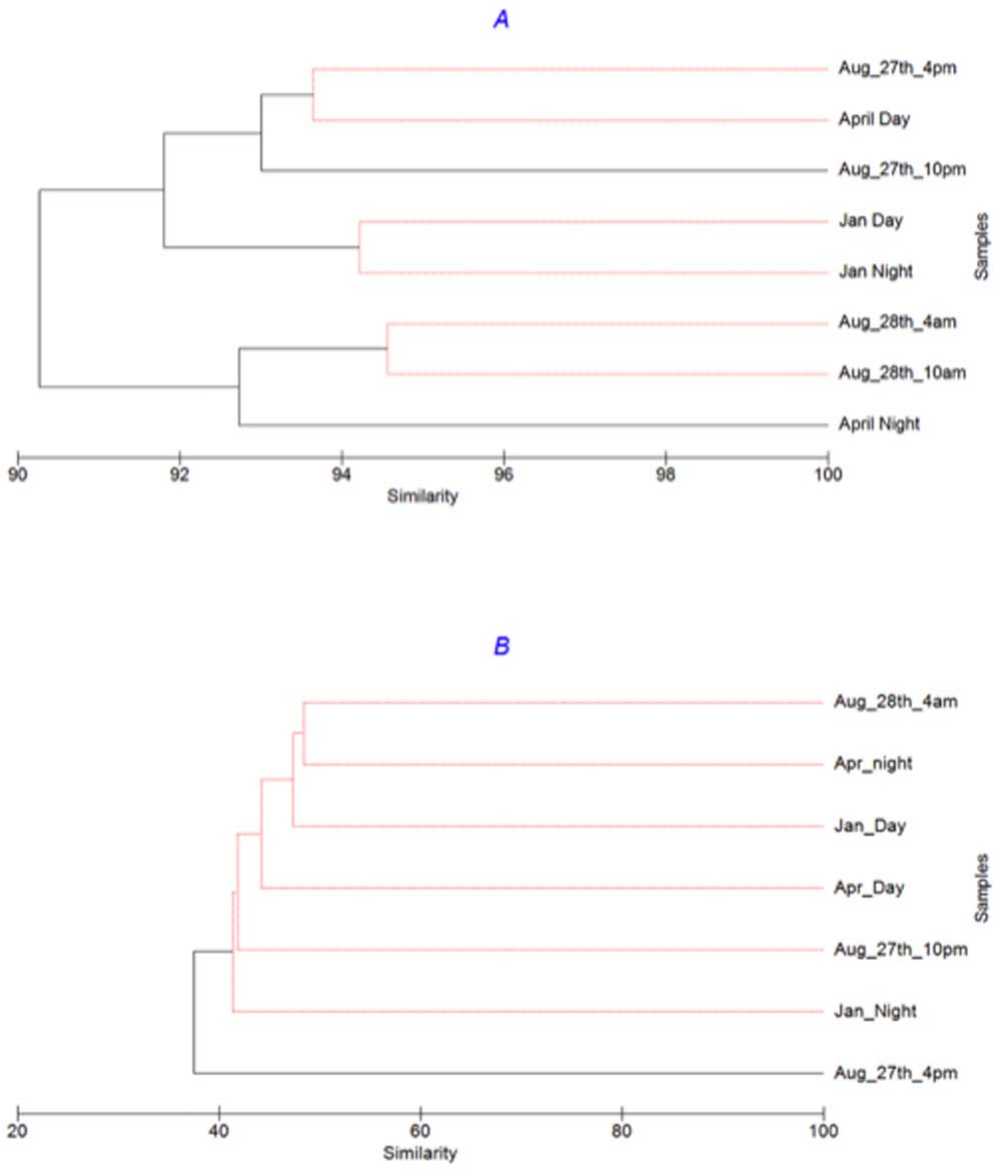
Group-average clustering dendrograms comparing (A) metagenomic sequences annotated against SEED subsystems; (B) metatranscriptomic sequences annotated against SEED subsystems.

To further explore potential proteins of unknown function and their contributions to the functioning of these communities, we compared dendrograms generated from all predicted ORFs (**Fig. 6**) to only those that could be annotated against the SEED database (**Fig. 9**) for both metagenomes and metatranscriptomes. It is obvious that the inclusion of unidentified predicted proteins caused a significant increase in the differences observed between samples. Strikingly, metagenomic sample similarity increased from an average of only ∼1% when comparing all predicted proteins (**Fig. 6A**) to >90% when comparing only sequences that can be annotated to a SEED subsystem (**Fig 9A**). For the metatranscriptomes, similarity still increased from an average of 17% (**Fig. 6B**) to ≥40% (**Fig 9B**).

In this study we tested the hypothesis that metagenomes and metatranscriptomes would track the overall diversity of 16S rRNA based community profiles and that they would show similar seasonal patterns. We found evidence to support this and interestingly, it is sets of unknown gene families that best distinguish among different samples through time. Detectable diversity is higher in winter, and at night within 24 hour periods, and this is potentially of particular relevance as there was more diversity in winter when nights are longer. Also, Lagrangian sampling demonstrates that even in complex communities with high diversity the robust temporal patterns of the marine microbial community observed in previous studies are still evident. Functional characterization using greater coverage in future studies should better elucidate these patterns. Using the Good's coverage estimates, we suggest that 20–30 time more sequencing effort than applied in this study (∼15 million sequences) is needed per metagenome, and 3–4 times more effort (1–1.5 million sequences) is needed per metatranscriptome, to provide equivalent coverage to the 16S rRNA V6 analysis.

This study demonstrates the potential of multi-omics to elucidate the diversity and functional potential of ecosystems. It also provides a powerful base for developing further hypotheses from which to launch future research. In the near future we will be increasing the resolution across time and space (e.g. inclusion of the E1 sampling location at 25 km off the coast – www.westernchannelobservatory.com), the sequencing depth of these studies, and the range of ‘omic’ technologies applied (e.g. meta-metabolomics), to help parameterise future metabolic models of this ecosystem.

## Supporting Information

Figure S1 **Dendrograms derived from16S rRNA V6 bacterial (A) and archaeal (B) community samples using group-average clustering of data and the Bray-Curtis similarity measure based on OTUs with >100 sequences.** Dendrograms derived from16S rRNA V6 bacterial (C) and archaeal (D) samples using group-average clustering of data using the Bray-Curtis similarity measure based on a presence-absence transformation of abundance data All samples were randomly-resampled to 4070 sequences. SIMPROF testing has been applied to branching structure: red lines indicate branches in which re-arrangement indicates no significant difference between communities.
(TIF)

Figure S2 **Putative transcript and gene richness as calculated from the metatranscriptomes and metagenomes.** Transcripts and genes sequences were translated into putative open reading frames (pORFs with >40 amino acids), all resulting fasta files were resampled to 66,529 sequences (smallest dataset – see Table 2) and then clustered at 95% amino acid identity over 80% length of fragment. The number of unique clusters is reported here.
(TIF)

Figure S3 **Principal component analysis of environmental variables demonstrating the seasonal differences in variables outlined in** **Table 3** **.**
(TIF)

Figure S4 **Good's coverage estimates against the percentage of metagenomic and metatranscriptomic sequences that could be annotated against a SEED subsystem (e-value <0.01).**
(TIF)

Table S1 **Bacterial and Archaeal 16S rRNA V6 specific primers used for amplification of the V6 region of 16S rRNA gene.** Lowercase base pairs indicate the 454-GS-FLX A (for forward primers) or B (for reverse primers) adapter. Primers originate from Huber et al. (2007)
(DOCX)

Table S2 **SIMPER analysis of the relative impact of different functional genes in providing differences between seasons for the metagenomic samples annotated against the Hierarchy 1 SEED subsystem database.** All data were randomly re-sampled prior to analysis and the abundances were transformed by square root. Jan – January; Aug – August; Av.Abund – square root of average abundance; Contrib% - individual % contribution of that metabolic function to the difference between samples; Cum.% - Cumulative % contribution of metabolic functions to difference between samples.
(DOCX)

Table S3 **SIMPER analysis of the relative impact of different functional genes in providing differences between day and night for each season for the metagenomic samples annotated against the Hierarchy 1 SEED subsystem database.** All data were randomly re-sampled prior to analysis and the abundances were transformed by square root. Jan – January; Aug – August; Av.Abund – square root of average abundance; Contrib% - individual % contribution of that metabolic function to the difference between samples; Cum.% - Cumulative % contribution of metabolic functions to difference between samples.
(DOCX)

Table S4 **SIMPER analysis of the contribution of functional genes to dissimilarities between seasons for the metagenomic samples annotated against the Hierarchy 1 SEED subsystem database.** All data were randomly re-sampled prior to analysis and the abundances were transformed by square root. Av.Abund – square root of average abundance; Contrib% - individual % contribution of that metabolic function to the dissimilarities between samples; Cum.% - Cumulative % contribution of metabolic functions to dissimilarities between samples.
(XLSX)

Table S5 **SIMPER analysis of the contribution of different functional genes to dissimilarities between day and night samples in January for the metagenomic samples annotated against the Hierarchy 1 SEED subsystem database.** All data were randomly re-sampled prior to analysis and the abundances were transformed by square root. Av.Abund – square root of average abundance; Contrib% - individual % contribution of that metabolic function to the dissimilarities between samples; Cum.% - Cumulative % contribution of metabolic functions to dissimilarities between samples.
(XLSX)

Table S6 **SIMPER analysis of the contribution of different functional genes to dissimilarities between day and night samples in April for the metagenomic samples annotated against the Hierarchy 1 SEED subsystem database.** All data were randomly re-sampled prior to analysis and the abundances were transformed by square root. Av.Abund – square root of average abundance; Contrib% - individual % contribution of that metabolic function to the dissimilarities between samples; Cum.% - Cumulative % contribution of metabolic functions to dissimilarities between samples.
(XLSX)

Table S7 **SIMPER analysis of the contribution of different functional genes to dissimilarities between day and night samples in August for the metagenomic samples annotated against the Hierarchy 1 SEED subsystem database.** All data were randomly re-sampled prior to analysis and the abundances were transformed by square root. Av.Abund – square root of average abundance; Contrib% - individual % contribution of that metabolic function to the dissimilarities between samples; Cum.% - Cumulative % contribution of metabolic functions to dissimilarities between samples.
(XLSX)

Table S8 **SIMPER analysis of the relative impact of different functional genes in providing differences between seasons for the metatranscriptomic samples annotated against the Hierarchy 1 SEED subsystem database.** All data were randomly re-sampled prior to analysis and the abundances were transformed by square root. Jan – January; Aug – August; Apr – April; Av.Abund – square root of average abundance; Contrib% - individual % contribution of that metabolic function to the difference between samples; Cum.% - Cumulative % contribution of metabolic functions to difference between samples.
(DOCX)

Table S9 **SIMPER analysis of the relative impact of different functional genes in providing differences between day and night for each season for the metatranscriptomic samples annotated against the Hierarchy 1 SEED subsystem database.** All data were randomly re-sampled prior to analysis and the abundances were transformed by square root. Jan – January; Aug – August; Av.Abund – square root of average abundance; Contrib% - individual % contribution of that metabolic function to the difference between samples; Cum.% - Cumulative % contribution of metabolic functions to difference between samples.
(DOCX)
