# Supplementary figures and images for: The Taxonomic and Functional Diversity of Microbes at a Temperate Coastal Site: A ‘Multi-Omic’ Study of Seasonal and Diel Temporal Variation
Source: PLoS One. 2010 Nov 29;5(11):e15545. doi: 10.1371/journal.pone.0015545 (PMC2993967; doi:10.1371/journal.pone.0015545)

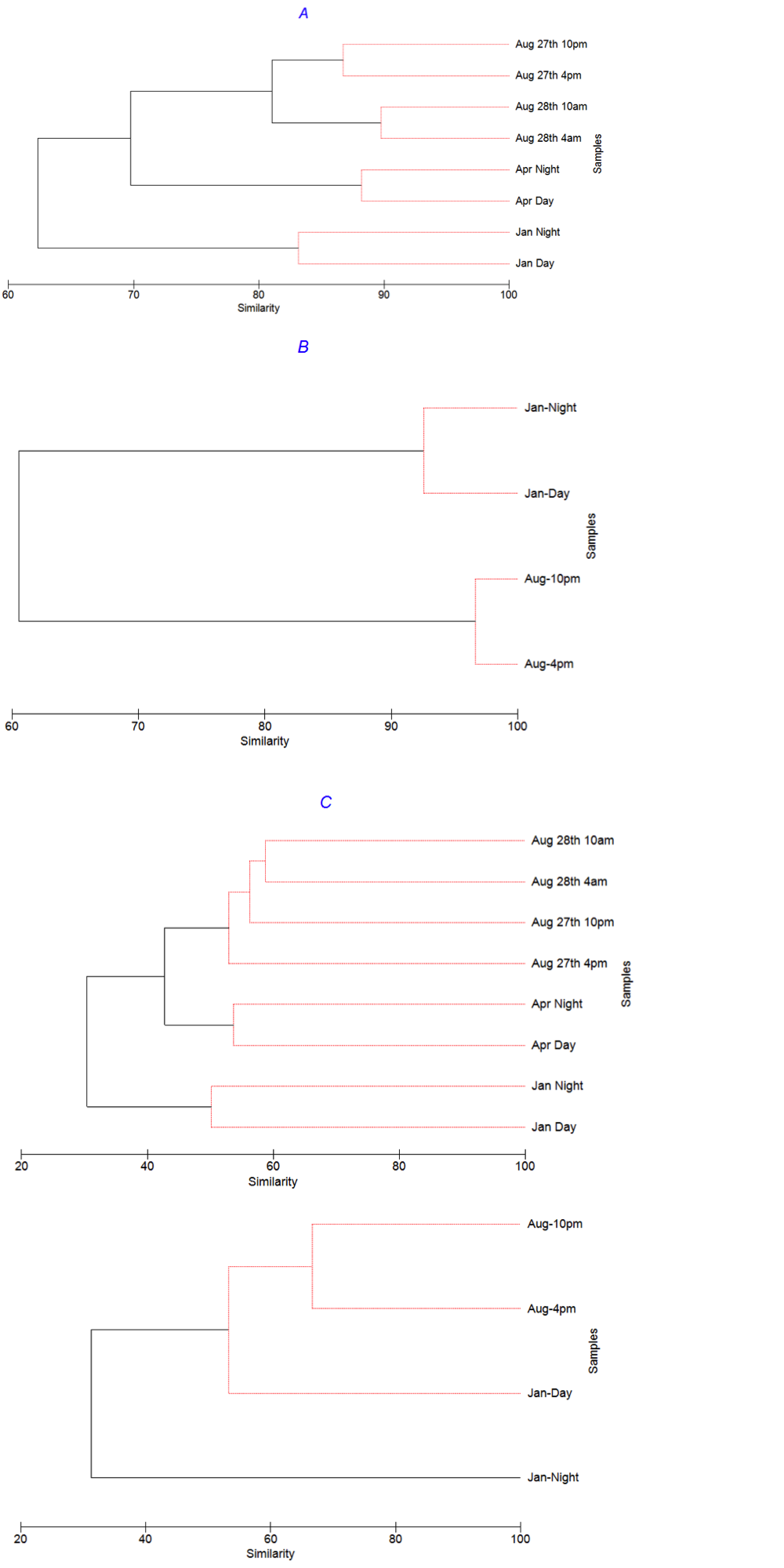

Supplement: Figure S1 — Dendrograms derived from16S rRNA V6 bacterial (A) and archaeal (B) community samples using group-average clustering of data and the Bray-Curtis similarity measure based on OTUs with >100 sequences. Dendrograms derived from16S rRNA V6 bacterial (C) and archaeal (D) samples using group-average clustering of data using the Bray-Curtis similarity measure based on a presence-absence transformation of abundance data All samples were randomly-resampled to 4070 sequences. SIMPROF testing has been applied to branching structure: red lines indicate branches in which re-arrangement indicates no significant difference between communities. (TIF) [file pone.0015545.s001.tif]

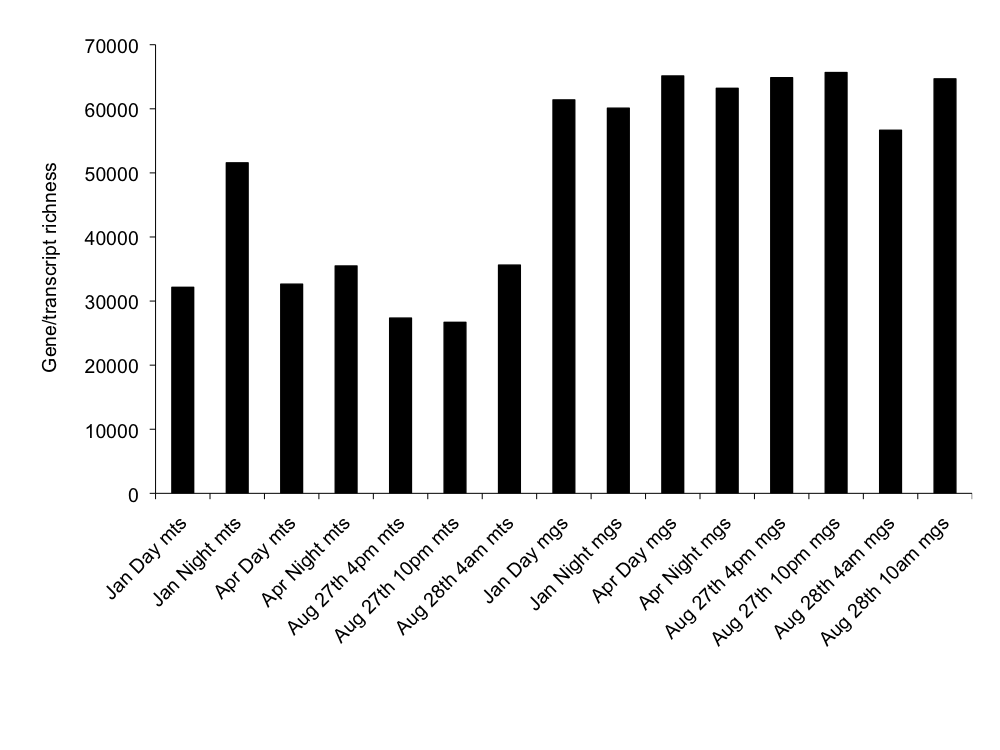

Supplement: Figure S2 — Putative transcript and gene richness as calculated from the metatranscriptomes and metagenomes. Transcripts and genes sequences were translated into putative open reading frames (pORFs with >40 amino acids), all resulting fasta files were resampled to 66,529 sequences (smallest dataset – see Table 2) and then clustered at 95% amino acid identity over 80% length of fragment. The number of unique clusters is reported here. (TIF) [file pone.0015545.s002.tif]

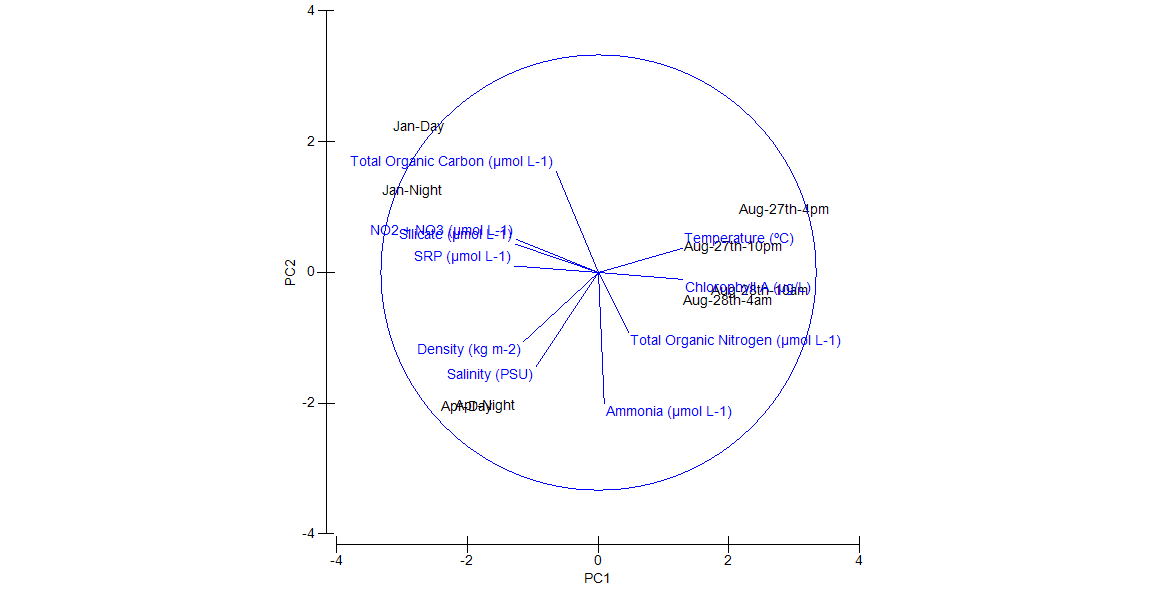

Supplement: Figure S3 — Principal component analysis of environmental variables demonstrating the seasonal differences in variables outlined in Table 3 . (TIF) [file pone.0015545.s003.tif]

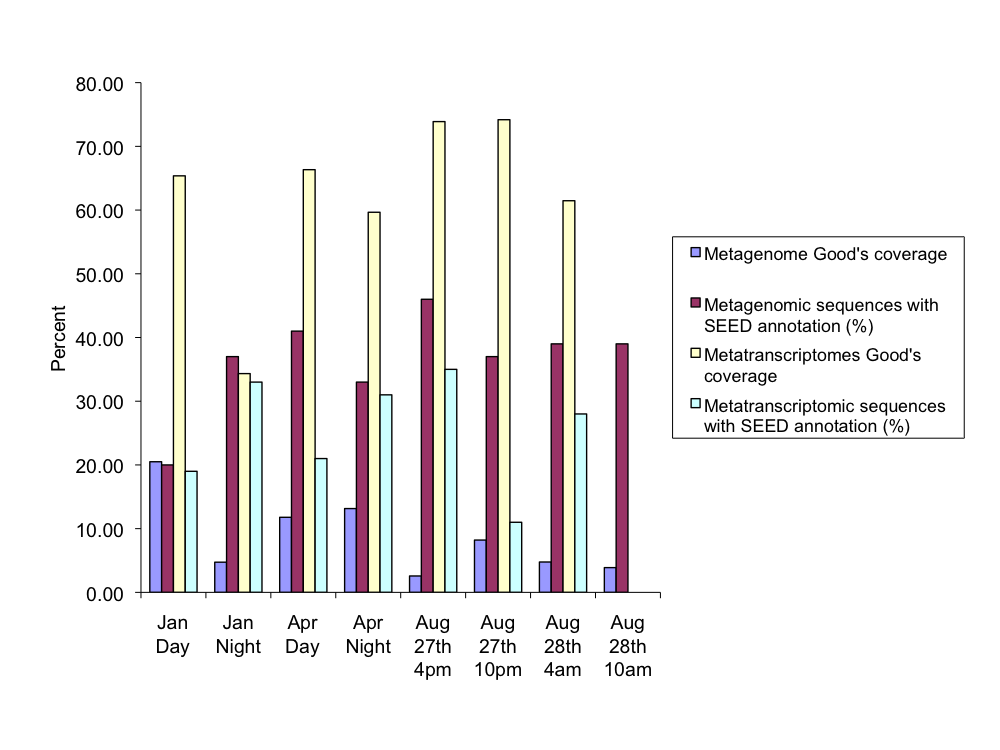

Supplement: Figure S4 — Good's coverage estimates against the percentage of metagenomic and metatranscriptomic sequences that could be annotated against a SEED subsystem (e-value <0.01). (TIF) [file pone.0015545.s004.tif]
