# Supplementary material for: The Taxonomic and Functional Diversity of Microbes at a Temperate Coastal Site: A ‘Multi-Omic’ Study of Seasonal and Diel Temporal Variation
Source: PLoS One. 2010 Nov 29;5(11):e15545. doi: 10.1371/journal.pone.0015545 (PMC2993967; doi:10.1371/journal.pone.0015545)
Supplement: Table S1 — Bacterial and Archaeal 16S rRNA V6 specific primers used for amplification of the V6 region of 16S rRNA gene. Lowercase base pairs indicate the 454-GS-FLX A (for forward primers) or B (for reverse primers) adapter. Primers originate from Huber et al. (2007) (DOCX) [file pone.0015545.s005.docx]

Table S1 – Bacterial and Archaeal 16S rRNA V6 specific primers used for amplification of the V6 region of 16S rRNA gene. Lowercase base pairs indicate the 454-GS-FLX A (for forward primers) or B (for reverse primers) adapter. Primers originate from Huber et al. (2007)

| 967F-PP | 5’-gcctccctcgcgccatcagCNACGCGAAGAACCTTANC-3’ |
| --- | --- |
| 967F-UC1 | 5’-gcctccctcgcgccatcagCAACGCGAAAAACCTTACC-3’ |
| 967F-UC2 | 5’-gcctccctcgcgccatcagCAACGCGCAGAACCTTACC-3’ |
| 967F-UC3 | 5’-gcctccctcgcgccatcagATACGCGARGAACCTTACC-3’ |
| 967F-AQ | 5’-gcctccctcgcgccatcagCTAACCGANGAACCTYACC-3’ |
| 1046R | 5’-gccttgccagcccgctcagCGACAGCCATGCANCACCT-3’ |
| 1046R-PP | 5’-gccttgccagcccgctcagCGACAACCATGCANCACCT-3’ |
| 1046R-AQ1 | 5’-gccttgccagcccgctcagCGACGGCCATGCANCACCT-3’ |
| 1046R-AQ2 | 5’-gccttgccagcccgctcagCGACGACCATGCANCACCT-3’ |
| 958arcF | 5’-gcctccctcgcgccatcagAATTGGANTCAACGCCGG-3’ |
| 1048arcR-major | 5’-gccttgccagcccgctcagCGRCGGCCATGCACCWC-3’ |
| 1048arcR-minor | 5’-gccttgccagcccgctcagCGRCRGCCATGYACCWC-3’ |
