# Supplementary material for: The Taxonomic and Functional Diversity of Microbes at a Temperate Coastal Site: A ‘Multi-Omic’ Study of Seasonal and Diel Temporal Variation
Source: PLoS One. 2010 Nov 29;5(11):e15545. doi: 10.1371/journal.pone.0015545 (PMC2993967; doi:10.1371/journal.pone.0015545)
Supplement: Table S2 — SIMPER analysis of the relative impact of different functional genes in providing differences between seasons for the metagenomic samples annotated against the Hierarchy 1 SEED subsystem database. All data were randomly re-sampled prior to analysis and the abundances were transformed by square root. Jan – January; Aug – August; Av.Abund – square root of average abundance; Contrib% - individual % contribution of that metabolic function to the difference between samples; Cum.% - Cumulative % contribution of metabolic functions to difference between samples. (DOCX) [file pone.0015545.s006.docx]

Table S2 – SIMPER analysis of the relative impact of different functional genes in providing differences **between seasons** for the **metagenomic** samples annotated against the Hierarchy 1 SEED subsystem database. All data were randomly re-sampled prior to analysis and the abundances were transformed by square root. Jan – January; Aug – August; Av.Abund – square root of average abundance; Contrib% - individual % contribution of that metabolic function to the difference between samples; Cum.% - Cumulative % contribution of metabolic functions to difference between samples.

|  | Group Jan | Group April |  |  |
| --- | --- | --- | --- | --- |
| Metabolic Function | Av.Abund | Av.Abund | Contrib% | Cum.% |
| Photosynthesis | 40.34 | 25.62 | 27.03 | 27.03 |
| Respiration | 82.44 | 74.25 | 9.74 | 36.77 |
| Clustering-based subsystems | 133.48 | 139.77 | 6.36 | 43.13 |
| Amino Acids and Derivatives | 113.68 | 113.95 | 4.05 | 47.19 |
| Carbohydrates | 116.74 | 119.8 | 3.85 | 51.03 |
| Regulation and Cell signaling | 34.08 | 34.25 | 3.21 | 54.24 |
| Virulence | 68.63 | 71.47 | 2.95 | 57.19 |
| Metabolism of Aromatic Compounds | 39.73 | 38.73 | 2.93 | 60.13 |
| Unclassified | 77.15 | 78.48 | 2.88 | 63.01 |
| Macromolecular Synthesis | 12.14 | 14.85 | 2.75 | 65.76 |
| Motility and Chemotaxis | 31.84 | 32.34 | 2.72 | 68.48 |
| Cofactors, Vitamins, Prosthetic Groups, Pigments | 89.89 | 91.63 | 2.61 | 71.09 |
| Membrane Transport | 50.38 | 51.01 | 2.58 | 73.67 |
| Nucleosides and Nucleotides | 70.6 | 68.11 | 2.52 | 76.19 |
| Cell Wall and Capsule | 74.14 | 75.42 | 2.4 | 78.59 |
| Secondary Metabolism | 10.95 | 8.63 | 2.35 | 80.94 |
| Sulfur Metabolism | 38.42 | 39.36 | 2.08 | 83.01 |
| Cell Division and Cell Cycle | 45.61 | 47.59 | 2 | 85.01 |
| RNA Metabolism | 70.78 | 68.82 | 1.99 | 87 |
| Protein Metabolism | 108.69 | 106.74 | 1.98 | 88.98 |
| Nitrogen Metabolism | 26.04 | 24.09 | 1.97 | 90.95 |
| Stress Response | 48.08 | 48.77 | 1.91 | 92.86 |
| Fatty Acids and Lipids | 45.17 | 43.44 | 1.75 | 94.62 |
| Miscellaneous | 19.34 | 20.87 | 1.55 | 96.17 |
| Phosphorus Metabolism | 41.84 | 42.84 | 1.3 | 97.47 |
| DNA Metabolism | 73.46 | 73.3 | 1.27 | 98.74 |
| Potassium metabolism | 24.88 | 24.66 | 0.63 | 99.37 |
| Prophage | 1.62 | 1.83 | 0.63 | 100 |
|  | Group Jan | Group Aug |  |  |
| Metabolic Function | Av.Abund | Av.Abund | Contrib% | Cum.% |
| Photosynthesis | 40.34 | 14 | 25.91 | 25.91 |
| Respiration | 82.44 | 72.31 | 10.47 | 36.38 |
| Virulence | 68.63 | 77.45 | 8.55 | 44.93 |
| Clustering-based subsystems | 133.48 | 140.34 | 6.64 | 51.57 |
| Amino Acids and Derivatives | 113.68 | 111.6 | 3.21 | 54.78 |
| Motility and Chemotaxis | 31.84 | 34.72 | 3.07 | 57.84 |
| Carbohydrates | 116.74 | 118.05 | 2.89 | 60.74 |
| Metabolism of Aromatic Compounds | 39.73 | 39.22 | 2.81 | 63.54 |
| Cell Wall and Capsule | 74.14 | 76.87 | 2.77 | 66.32 |
| Potassium metabolism | 24.88 | 27.48 | 2.65 | 68.96 |
| Unclassified | 77.15 | 77.48 | 2.61 | 71.57 |
| Membrane Transport | 50.38 | 48.91 | 2.58 | 74.15 |
| Cofactors, Vitamins, Prosthetic Groups, Pigments | 89.89 | 90.76 | 2.32 | 76.47 |
| Sulfur Metabolism | 38.42 | 36.47 | 2.2 | 78.67 |
| Stress Response | 48.08 | 49.84 | 2.18 | 80.85 |
| Phosphorus Metabolism | 41.84 | 44.02 | 2.1 | 82.96 |
| DNA Metabolism | 73.46 | 75.35 | 2.08 | 85.03 |
| Regulation and Cell signaling | 34.08 | 34.75 | 2.04 | 87.07 |
| Nucleosides and Nucleotides | 70.6 | 68.8 | 1.78 | 88.85 |
| Secondary Metabolism | 10.95 | 9.28 | 1.62 | 90.47 |
| Cell Division and Cell Cycle | 45.61 | 47.12 | 1.46 | 91.93 |
| Protein Metabolism | 108.69 | 107.23 | 1.42 | 93.35 |
| Fatty Acids and Lipids | 45.17 | 44.37 | 1.32 | 94.67 |
| Miscellaneous | 19.34 | 20.67 | 1.29 | 95.96 |
| RNA Metabolism | 70.78 | 70 | 1.22 | 97.18 |
| Macromolecular Synthesis | 12.14 | 12.25 | 1.16 | 98.34 |
| Nitrogen Metabolism | 26.04 | 26.22 | 0.82 | 99.16 |
| Prophage | 1.62 | 1.29 | 0.6 | 99.76 |
| Dormancy and Sporulation | 0 | 0.25 | 0.24 | 100 |
|  | Group April | Group Aug |  |  |
| Metabolic Function | Av.Abund | Av.Abund | Contrib% | Cum.% |
| Photosynthesis | 25.62 | 14 | 18.34 | 18.34 |
| Virulence | 71.47 | 77.45 | 9.54 | 27.88 |
| Amino Acids and Derivatives | 113.95 | 111.6 | 5.75 | 33.63 |
| Motility and Chemotaxis | 32.34 | 34.72 | 4.71 | 38.35 |
| Regulation and Cell signaling | 34.25 | 34.75 | 4.37 | 42.72 |
| Unclassified | 78.48 | 77.48 | 4.24 | 46.96 |
| Macromolecular Synthesis | 14.85 | 12.25 | 4.24 | 51.2 |
| Membrane Transport | 51.01 | 48.91 | 4.16 | 55.36 |
| Sulfur Metabolism | 39.36 | 36.47 | 4.07 | 59.44 |
| Cell Wall and Capsule | 75.42 | 76.87 | 3.99 | 63.43 |
| Carbohydrates | 119.8 | 118.05 | 3.96 | 67.39 |
| Potassium metabolism | 24.66 | 27.48 | 3.96 | 71.35 |
| DNA Metabolism | 73.3 | 75.35 | 3.38 | 74.74 |
| Respiration | 74.25 | 72.31 | 2.98 | 77.71 |
| Nitrogen Metabolism | 24.09 | 26.22 | 2.94 | 80.65 |
| Clustering-based subsystems | 139.77 | 140.34 | 2.23 | 82.88 |
| Phosphorus Metabolism | 42.84 | 44.02 | 2.18 | 85.07 |
| Cofactors, Vitamins, Prosthetic Groups, Pigments | 91.63 | 90.76 | 2.02 | 87.09 |
| Stress Response | 48.77 | 49.84 | 2 | 89.09 |
| RNA Metabolism | 68.82 | 70 | 1.81 | 90.9 |
| Metabolism of Aromatic Compounds | 38.73 | 39.22 | 1.67 | 92.57 |
| Protein Metabolism | 106.74 | 107.23 | 1.37 | 93.94 |
| Fatty Acids and Lipids | 43.44 | 44.37 | 1.28 | 95.22 |
| Nucleosides and Nucleotides | 68.11 | 68.8 | 1.1 | 96.32 |
| Secondary Metabolism | 8.63 | 9.28 | 1.07 | 97.39 |
| Prophage | 1.83 | 1.29 | 0.85 | 98.25 |
| Miscellaneous | 20.87 | 20.67 | 0.77 | 99.02 |
| Cell Division and Cell Cycle | 47.59 | 47.12 | 0.64 | 99.66 |
| Dormancy and Sporulation | 0 | 0.25 | 0.34 | 100 |
