# Supplementary material for: The Taxonomic and Functional Diversity of Microbes at a Temperate Coastal Site: A ‘Multi-Omic’ Study of Seasonal and Diel Temporal Variation
Source: PLoS One. 2010 Nov 29;5(11):e15545. doi: 10.1371/journal.pone.0015545 (PMC2993967; doi:10.1371/journal.pone.0015545)
Supplement: Table S3 — SIMPER analysis of the relative impact of different functional genes in providing differences between day and night for each season for the metagenomic samples annotated against the Hierarchy 1 SEED subsystem database. All data were randomly re-sampled prior to analysis and the abundances were transformed by square root. Jan – January; Aug – August; Av.Abund – square root of average abundance; Contrib% - individual % contribution of that metabolic function to the difference between samples; Cum.% - Cumulative % contribution of metabolic functions to difference between samples. (DOCX) [file pone.0015545.s007.docx]

Table S3 – SIMPER analysis of the relative impact of different functional genes in providing differences **between day and night** for each season for the **metagenomic** samples annotated against the Hierarchy 1 SEED subsystem database. All data were randomly re-sampled prior to analysis and the abundances were transformed by square root. Jan – January; Aug – August; Av.Abund – square root of average abundance; Contrib% - individual % contribution of that metabolic function to the difference between samples; Cum.% - Cumulative % contribution of metabolic functions to difference between samples.

| **January**  Metabolic Function | Day  Av.Abund | Night  Av.Abund | Contrib% | Cum.% |
| --- | --- | --- | --- | --- |
| Photosynthesis | 14.56 | 66.13 | 40.92 | 40.92 |
| Respiration | 72.79 | 92.09 | 15.31 | 56.23 |
| Metabolism of Aromatic Compounds | 42.63 | 36.84 | 4.59 | 60.82 |
| Amino Acids and Derivatives | 116.25 | 111.11 | 4.08 | 64.9 |
| Carbohydrates | 119.08 | 114.4 | 3.72 | 68.62 |
| Cofactors, Vitamins, Prosthetic Groups, Pigments | 92.18 | 87.61 | 3.63 | 72.25 |
| Membrane Transport | 52.57 | 48.19 | 3.48 | 75.73 |
| Stress Response | 49.97 | 46.18 | 3 | 78.73 |
| Unclassified | 78.96 | 75.35 | 2.87 | 81.6 |
| Sulfur Metabolism | 40.05 | 36.8 | 2.58 | 84.18 |
| Fatty Acids and Lipids | 46.53 | 43.81 | 2.16 | 86.34 |
| Nucleosides and Nucleotides | 71.78 | 69.43 | 1.87 | 88.21 |
| Cell Wall and Capsule | 75.05 | 73.23 | 1.44 | 89.65 |
| Clustering-based subsystems | 134.35 | 132.61 | 1.38 | 91.03 |
| RNA Metabolism | 71.64 | 69.92 | 1.37 | 92.4 |
| Prophage | 1 | 2.24 | 0.98 | 93.38 |
| Cell Division and Cell Cycle | 46.22 | 45.01 | 0.96 | 94.34 |
| Potassium metabolism | 25.46 | 24.31 | 0.91 | 95.25 |
| Phosphorus Metabolism | 42.38 | 41.3 | 0.85 | 96.1 |
| Regulation and Cell signaling | 34.58 | 33.57 | 0.8 | 96.9 |
| Protein Metabolism | 109.14 | 108.25 | 0.71 | 97.61 |
| DNA Metabolism | 73.9 | 73.01 | 0.7 | 98.32 |
| Motility and Chemotaxis | 32.16 | 31.53 | 0.5 | 98.81 |
| Nitrogen Metabolism | 25.85 | 26.23 | 0.3 | 99.12 |
| Secondary Metabolism | 10.77 | 11.14 | 0.29 | 99.41 |
| Miscellaneous | 19.52 | 19.16 | 0.29 | 99.7 |
| Virulence | 68.45 | 68.8 | 0.27 | 99.97 |
| Macromolecular Synthesis | 12.17 | 12.12 | 0.03 | 100 |
| **April**  Metabolic Function | Day  Av.Abund | Night  Av.Abund | Contrib% | Cum.% |
| Photosynthesis | 12.65 | 38.59 | 27.23 | 27.23 |
| Amino Acids and Derivatives | 117.95 | 109.95 | 8.4 | 35.64 |
| Regulation and Cell signalling | 37.42 | 31.08 | 6.65 | 42.29 |
| Virulence | 68.64 | 74.3 | 5.95 | 48.24 |
| Motility and Chemotaxis | 29.65 | 35.03 | 5.65 | 53.89 |
| Unclassified | 81.04 | 75.91 | 5.38 | 59.27 |
| Cell Wall and Capsule | 73.05 | 77.79 | 4.98 | 64.25 |
| Membrane Transport | 53.28 | 48.73 | 4.78 | 69.02 |
| Carbohydrates | 122 | 117.6 | 4.62 | 73.65 |
| Macromolecular Synthesis | 16.94 | 12.77 | 4.38 | 78.03 |
| Sulphur Metabolism | 40.9 | 37.82 | 3.24 | 81.27 |
| DNA Metabolism | 72.05 | 74.56 | 2.64 | 83.9 |
| Cofactors, Vitamins, Prosthetic Groups, Pigments | 90.49 | 92.77 | 2.39 | 86.3 |
| Phosphorus Metabolism | 41.8 | 43.89 | 2.19 | 88.49 |
| Secondary Metabolism | 7.94 | 9.33 | 1.46 | 89.95 |
| Protein Metabolism | 106.08 | 107.4 | 1.38 | 91.33 |
| RNA Metabolism | 68.26 | 69.38 | 1.17 | 92.5 |
| Potassium metabolism | 24.21 | 25.12 | 0.96 | 93.45 |
| Nucleosides and Nucleotides | 68.56 | 67.66 | 0.95 | 94.4 |
| Stress Response | 48.35 | 49.18 | 0.87 | 95.27 |
| Prophage | 1.41 | 2.24 | 0.86 | 96.14 |
| Clustering-based subsystems | 139.37 | 140.17 | 0.83 | 96.97 |
| Respiration | 73.86 | 74.63 | 0.81 | 97.78 |
| Metabolism of Aromatic Compounds | 38.38 | 39.08 | 0.73 | 98.52 |
| Miscellaneous | 21.17 | 20.57 | 0.63 | 99.15 |
| Cell Division and Cell Cycle | 47.81 | 47.36 | 0.47 | 99.62 |
| Nitrogen Metabolism | 24.23 | 23.96 | 0.28 | 99.9 |
| Fatty Acids and Lipids | 43.39 | 43.49 | 0.1 | 100 |
| Dormancy and Sporulation | 0 | 0 | 0 | 100 |
| **August**  Metabolic Function | Day  Av.Abund | Night  Av.Abund | Contrib% | Cum.% |
| Virulence | 73.82 | 81.09 | 10.92 | 10.92 |
| Unclassified | 79.63 | 75.33 | 6.47 | 17.4 |
| Regulation and Cell signalling | 36.85 | 32.65 | 6.31 | 23.71 |
| Amino Acids and Derivatives | 113.56 | 109.64 | 5.89 | 29.6 |
| Cell Wall and Capsule | 74.92 | 78.82 | 5.85 | 35.45 |
| Respiration | 74.2 | 70.43 | 5.66 | 41.12 |
| Carbohydrates | 119.84 | 116.27 | 5.37 | 46.49 |
| Motility and Chemotaxis | 32.97 | 36.46 | 5.25 | 51.73 |
| Membrane Transport | 50.56 | 47.27 | 4.95 | 56.68 |
| Clustering-based subsystems | 138.74 | 141.94 | 4.81 | 61.49 |
| Photosynthesis | 12.65 | 15.34 | 4.04 | 65.53 |
| Potassium metabolism | 26.19 | 28.77 | 3.87 | 69.4 |
| Macromolecular Synthesis | 13.44 | 11.05 | 3.59 | 72.99 |
| DNA Metabolism | 74.28 | 76.42 | 3.21 | 76.21 |
| RNA Metabolism | 69.12 | 70.88 | 2.65 | 78.86 |
| Nitrogen Metabolism | 27.06 | 25.38 | 2.54 | 81.39 |
| Cofactors, Vitamins, Prosthetic Groups, Pigments | 90.47 | 91.04 | 2.4 | 83.79 |
| Stress Response | 49.13 | 50.56 | 2.27 | 86.06 |
| Metabolism of Aromatic Compounds | 38.97 | 39.47 | 2.15 | 88.21 |
| Phosphorus Metabolism | 43.53 | 44.5 | 2.15 | 90.35 |
| Sulphur Metabolism | 36.62 | 36.31 | 2.11 | 92.47 |
| Protein Metabolism | 107.4 | 107.06 | 1.77 | 94.24 |
| Nucleosides and Nucleotides | 69.24 | 68.36 | 1.33 | 95.56 |
| Miscellaneous | 20.77 | 20.57 | 1.11 | 96.68 |
| Prophage | 1.57 | 1 | 0.86 | 97.54 |
| Fatty Acids and Lipids | 44.61 | 44.12 | 0.77 | 98.31 |
| Dormancy and Sporulation | 0.5 | 0 | 0.75 | 99.06 |
| Cell Division and Cell Cycle | 47.3 | 46.95 | 0.54 | 99.6 |
| Secondary Metabolism | 9.38 | 9.19 | 0.4 | 100 |
