# Supplementary material for: The Taxonomic and Functional Diversity of Microbes at a Temperate Coastal Site: A ‘Multi-Omic’ Study of Seasonal and Diel Temporal Variation
Source: PLoS One. 2010 Nov 29;5(11):e15545. doi: 10.1371/journal.pone.0015545 (PMC2993967; doi:10.1371/journal.pone.0015545)
Supplement: Table S8 — SIMPER analysis of the relative impact of different functional genes in providing differences between seasons for the metatranscriptomic samples annotated against the Hierarchy 1 SEED subsystem database. All data were randomly re-sampled prior to analysis and the abundances were transformed by square root. Jan – January; Aug – August; Apr – April; Av.Abund – square root of average abundance; Contrib% - individual % contribution of that metabolic function to the difference between samples; Cum.% - Cumulative % contribution of metabolic functions to difference between samples. (DOCX) [file pone.0015545.s012.docx]

Table S8 – SIMPER analysis of the relative impact of different functional genes in providing differences **between seasons** for the **metatranscriptomic** samples annotated against the Hierarchy 1 SEED subsystem database. All data were randomly re-sampled prior to analysis and the abundances were transformed by square root. Jan – January; Aug – August; Apr – April; Av.Abund – square root of average abundance; Contrib% - individual % contribution of that metabolic function to the difference between samples; Cum.% - Cumulative % contribution of metabolic functions to difference between samples.

|  | Group Apr | Group Jan |  |  |
| --- | --- | --- | --- | --- |
| Metabolic Function | Av.Abund | Av.Abund | Contrib% | Cum.% |
| RNA Metabolism | 23.67 | 17.14 | 11.42 | 11.42 |
| Unclassified | 13.66 | 8.64 | 8.6 | 20.02 |
| Motility and Chemotaxis | 9.93 | 2.37 | 8.41 | 28.43 |
| Carbohydrates | 19.79 | 25.15 | 6.94 | 35.37 |
| Virulence | 15.83 | 14.2 | 5.98 | 41.35 |
| Membrane Transport | 9.01 | 3.67 | 5.93 | 47.29 |
| Regulation and Cell signalling | 7.54 | 5.93 | 5.16 | 52.45 |
| Protein Metabolism | 12.34 | 11.17 | 5.11 | 57.55 |
| Clustering-based subsystems | 12.76 | 16.67 | 4.32 | 61.87 |
| Amino Acids and Derivatives | 9.81 | 13.45 | 4.06 | 65.93 |
| Cofactors, Vitamins, Prosthetic Groups, Pigments | 9.25 | 12.52 | 3.88 | 69.81 |
| Stress Response | 7.4 | 7.34 | 3.34 | 73.16 |
| Respiration | 5.08 | 6.76 | 3.08 | 76.24 |
| Cell Wall and Capsule | 10.95 | 11.26 | 2.87 | 79.11 |
| Nucleosides and Nucleotides | 5.09 | 7.68 | 2.82 | 81.92 |
| Photosynthesis | 1.62 | 2.55 | 2.78 | 84.7 |
| Cell Division and Cell Cycle | 5.33 | 7.43 | 2.46 | 87.17 |
| Fatty Acids and Lipids | 1.91 | 3.92 | 2.39 | 89.55 |
| Secondary Metabolism | 0 | 2.08 | 2.26 | 91.81 |
| Metabolism of Aromatic Compounds | 2.32 | 3.16 | 1.89 | 93.71 |
| Miscellaneous | 5.97 | 5.36 | 1.13 | 94.84 |
| Nitrogen Metabolism | 1 | 1 | 1.1 | 95.94 |
| DNA Metabolism | 6.42 | 6.58 | 0.94 | 96.89 |
| Potassium metabolism | 1.5 | 2.32 | 0.9 | 97.79 |
| Macromolecular Synthesis | 0.71 | 0 | 0.79 | 98.58 |
| Phosphorus Metabolism | 6.09 | 6.78 | 0.75 | 99.32 |
| Sulfur Metabolism | 3.05 | 3.59 | 0.68 | 100 |
|  | Group Apr | Group Aug |  |  |
| Metabolic Function | Av.Abund | Av.Abund | Contrib% | Cum.% |
| Membrane Transport | 9.01 | 17.38 | 13.06 | 13.06 |
| Unclassified | 13.66 | 6.75 | 8.68 | 21.74 |
| RNA Metabolism | 23.67 | 25.63 | 7.77 | 29.52 |
| Motility and Chemotaxis | 9.93 | 4.42 | 6.77 | 36.29 |
| Cofactors, Vitamins, Prosthetic Groups, Pigments | 9.25 | 6.31 | 6.57 | 42.86 |
| Protein Metabolism | 12.34 | 6.86 | 5.66 | 48.52 |
| Regulation and Cell signaling | 7.54 | 3.79 | 4.7 | 53.22 |
| Miscellaneous | 5.97 | 9.76 | 4.67 | 57.89 |
| Amino Acids and Derivatives | 9.81 | 8.77 | 4.65 | 62.54 |
| Carbohydrates | 19.79 | 21.64 | 4.57 | 67.11 |
| Stress Response | 7.4 | 3.9 | 4.13 | 71.25 |
| Clustering-based subsystems | 12.76 | 9.43 | 3.95 | 75.19 |
| Virulence | 15.83 | 14.75 | 3.39 | 78.58 |
| Phosphorus Metabolism | 6.09 | 3.45 | 2.68 | 81.26 |
| DNA Metabolism | 6.42 | 4.24 | 2.65 | 83.91 |
| Cell Wall and Capsule | 10.95 | 8.84 | 2.2 | 86.11 |
| Nucleosides and Nucleotides | 5.09 | 3.19 | 1.97 | 88.08 |
| Respiration | 5.08 | 5.97 | 1.91 | 89.98 |
| Photosynthesis | 1.62 | 0 | 1.64 | 91.62 |
| Potassium metabolism | 1.5 | 0 | 1.51 | 93.13 |
| Cell Division and Cell Cycle | 5.33 | 6.73 | 1.43 | 94.56 |
| Metabolism of Aromatic Compounds | 2.32 | 1.82 | 1.31 | 95.86 |
| Fatty Acids and Lipids | 1.91 | 1.69 | 1.27 | 97.13 |
| Sulfur Metabolism | 3.05 | 2.39 | 0.99 | 98.12 |
| Nitrogen Metabolism | 1 | 1.72 | 0.72 | 98.85 |
| Macromolecular Synthesis | 0.71 | 0.47 | 0.72 | 99.56 |
| Secondary Metabolism | 0 | 0.47 | 0.44 | 100 |
|  | Group Jan | Group Aug |  |  |
| Metabolic Function | Av.Abund | Av.Abund | Contrib% | Cum.% |
| Membrane Transport | 3.67 | 17.38 | 12.77 | 12.77 |
| RNA Metabolism | 17.14 | 25.63 | 10.91 | 23.69 |
| Cofactors, Vitamins, Prosthetic Groups, Pigments | 12.52 | 6.31 | 6.9 | 30.59 |
| Clustering-based subsystems | 16.67 | 9.43 | 6.43 | 37.02 |
| Amino Acids and Derivatives | 13.45 | 8.77 | 5.45 | 42.47 |
| Carbohydrates | 25.15 | 21.64 | 4.82 | 47.28 |
| Virulence | 14.2 | 14.75 | 4.78 | 52.07 |
| Miscellaneous | 5.36 | 9.76 | 4.33 | 56.4 |
| Nucleosides and Nucleotides | 7.68 | 3.19 | 3.95 | 60.35 |
| Unclassified | 8.64 | 6.75 | 3.88 | 64.23 |
| Protein Metabolism | 11.17 | 6.86 | 3.87 | 68.1 |
| Stress Response | 7.34 | 3.9 | 3.09 | 71.19 |
| Phosphorus Metabolism | 6.78 | 3.45 | 2.92 | 74.11 |
| Cell Wall and Capsule | 11.26 | 8.84 | 2.87 | 76.98 |
| Motility and Chemotaxis | 2.37 | 4.42 | 2.59 | 79.57 |
| Respiration | 6.76 | 5.97 | 2.38 | 81.95 |
| DNA Metabolism | 6.58 | 4.24 | 2.28 | 84.23 |
| Photosynthesis | 2.55 | 0 | 2.17 | 86.4 |
| Fatty Acids and Lipids | 3.92 | 1.69 | 2.05 | 88.45 |
| Potassium metabolism | 2.32 | 0 | 2.03 | 90.48 |
| Metabolism of Aromatic Compounds | 3.16 | 1.82 | 1.93 | 92.41 |
| Regulation and Cell signaling | 5.93 | 3.79 | 1.93 | 94.34 |
| Cell Division and Cell Cycle | 7.43 | 6.73 | 1.73 | 96.06 |
| Secondary Metabolism | 2.08 | 0.47 | 1.53 | 97.59 |
| Sulfur Metabolism | 3.59 | 2.39 | 1.09 | 98.68 |
| Nitrogen Metabolism | 1 | 1.72 | 0.89 | 99.57 |
| Macromolecular Synthesis | 0 | 0.47 | 0.43 | 100 |
