# Supplementary material for: The Taxonomic and Functional Diversity of Microbes at a Temperate Coastal Site: A ‘Multi-Omic’ Study of Seasonal and Diel Temporal Variation
Source: PLoS One. 2010 Nov 29;5(11):e15545. doi: 10.1371/journal.pone.0015545 (PMC2993967; doi:10.1371/journal.pone.0015545)
Supplement: Table S9 — SIMPER analysis of the relative impact of different functional genes in providing differences between day and night for each season for the metatranscriptomic samples annotated against the Hierarchy 1 SEED subsystem database. All data were randomly re-sampled prior to analysis and the abundances were transformed by square root. Jan – January; Aug – August; Av.Abund – square root of average abundance; Contrib% - individual % contribution of that metabolic function to the difference between samples; Cum.% - Cumulative % contribution of metabolic functions to difference between samples. (DOCX) [file pone.0015545.s013.docx]

Table S9 – SIMPER analysis of the relative impact of different functional genes in providing differences **between day and night** for each season for the **metatranscriptomic** samples annotated against the Hierarchy 1 SEED subsystem database. All data were randomly re-sampled prior to analysis and the abundances were transformed by square root. Jan – January; Aug – August; Av.Abund – square root of average abundance; Contrib% - individual % contribution of that metabolic function to the difference between samples; Cum.% - Cumulative % contribution of metabolic functions to difference between samples.

| Metabolic Function | January Night | January Day | Contrib% | Cum.% |
| --- | --- | --- | --- | --- |
| RNA Metabolism | 8.37 | 25.9 | 17.05 | 17.05 |
| Virulence | 19.67 | 8.72 | 10.65 | 27.71 |
| Protein Metabolism | 7.68 | 14.66 | 6.79 | 34.5 |
| Carbohydrates | 28.6 | 21.7 | 6.71 | 41.21 |
| Unclassified | 5.2 | 12.08 | 6.7 | 47.9 |
| Cell Wall and Capsule | 13.86 | 8.66 | 5.05 | 52.96 |
| Photosynthesis | 0 | 5.1 | 4.96 | 57.92 |
| Respiration | 4.24 | 9.27 | 4.89 | 62.81 |
| Amino Acids and Derivatives | 15.81 | 11.09 | 4.59 | 67.4 |
| Cofactors, Vitamins, Prosthetic Groups, Pigments | 14.8 | 10.25 | 4.43 | 71.83 |
| Cell Division and Cell Cycle | 9.38 | 5.48 | 3.8 | 75.62 |
| Metabolism of Aromatic Compounds | 1.41 | 4.9 | 3.39 | 79.01 |
| Fatty Acids and Lipids | 2.45 | 5.39 | 2.85 | 81.87 |
| Motility and Chemotaxis | 1 | 3.74 | 2.67 | 84.53 |
| Nucleosides and Nucleotides | 6.48 | 8.89 | 2.34 | 86.87 |
| Secondary Metabolism | 1 | 3.16 | 2.1 | 88.98 |
| Membrane Transport | 2.65 | 4.69 | 1.99 | 90.96 |
| Nitrogen Metabolism | 0 | 2 | 1.94 | 92.91 |
| Regulation and Cell signaling | 6.86 | 5 | 1.8 | 94.71 |
| Miscellaneous | 6.24 | 4.47 | 1.72 | 96.44 |
| DNA Metabolism | 6.16 | 7 | 0.81 | 97.25 |
| Stress Response | 7.75 | 6.93 | 0.8 | 98.05 |
| Clustering-based subsystems | 16.34 | 17 | 0.64 | 98.69 |
| Potassium metabolism | 2 | 2.65 | 0.63 | 99.32 |
| Sulfur Metabolism | 3.87 | 3.32 | 0.54 | 99.86 |
| Phosphorus Metabolism | 6.71 | 6.86 | 0.14 | 100 |
| Macromolecular Synthesis | 0 | 0 | 0 | 100 |
| Metabolic Function | April Night | April Day | Contrib% | Cum.% |
| Unclassified | 6.63 | 20.69 | 14.39 | 14.39 |
| RNA Metabolism | 18.49 | 28.84 | 10.59 | 24.98 |
| Regulation and Cell signalling | 12.25 | 2.83 | 9.64 | 34.62 |
| Protein Metabolism | 17 | 7.68 | 9.54 | 44.16 |
| Motility and Chemotaxis | 5.66 | 14.21 | 8.76 | 52.92 |
| Carbohydrates | 23.39 | 16.19 | 7.37 | 60.29 |
| Virulence | 19.05 | 12.61 | 6.59 | 66.88 |
| Stress Response | 10.44 | 4.36 | 6.22 | 73.11 |
| Membrane Transport | 7.07 | 10.95 | 3.97 | 77.08 |
| Clustering-based subsystems | 14.42 | 11.09 | 3.41 | 80.49 |
| Respiration | 6.56 | 3.61 | 3.02 | 83.51 |
| Cofactors, Vitamins, Prosthetic Groups, Pigments | 10.63 | 7.87 | 2.82 | 86.33 |
| Fatty Acids and Lipids | 2.83 | 1 | 1.87 | 88.2 |
| DNA Metabolism | 7.28 | 5.57 | 1.75 | 89.96 |
| Macromolecular Synthesis | 0 | 1.41 | 1.45 | 91.4 |
| Photosynthesis | 1 | 2.24 | 1.27 | 92.67 |
| Phosphorus Metabolism | 5.48 | 6.71 | 1.26 | 93.93 |
| Miscellaneous | 6.56 | 5.39 | 1.2 | 95.13 |
| Potassium metabolism | 2 | 1 | 1.02 | 96.15 |
| Amino Acids and Derivatives | 9.38 | 10.25 | 0.89 | 97.04 |
| Sulfur Metabolism | 2.65 | 3.46 | 0.84 | 97.88 |
| Cell Division and Cell Cycle | 5.66 | 5 | 0.67 | 98.55 |
| Metabolism of Aromatic Compounds | 2 | 2.65 | 0.66 | 99.21 |
| Nucleosides and Nucleotides | 4.8 | 5.39 | 0.6 | 99.81 |
| Cell Wall and Capsule | 10.86 | 11.05 | 0.19 | 100 |
| Metabolic Function | August Day | August Night | Contrib% | Cum.% |
| Membrane Transport | 35.07 | 8.54 | 23.52 | 23.52 |
| Miscellaneous | 17.83 | 5.73 | 10.61 | 34.13 |
| RNA Metabolism | 19.85 | 28.52 | 8.02 | 42.16 |
| Carbohydrates | 15.84 | 24.54 | 7.66 | 49.81 |
| Amino Acids and Derivatives | 3.46 | 11.42 | 6.71 | 56.53 |
| Unclassified | 2.24 | 9 | 5.78 | 62.31 |
| Cofactors, Vitamins, Prosthetic Groups, Pigments | 2.65 | 8.15 | 5.26 | 67.57 |
| Clustering-based subsystems | 6.56 | 10.87 | 3.65 | 71.23 |
| Virulence | 12.17 | 16.05 | 3.46 | 74.69 |
| Motility and Chemotaxis | 1.73 | 5.77 | 3.33 | 78.02 |
| Stress Response | 1.41 | 5.14 | 3.19 | 81.21 |
| Metabolism of Aromatic Compounds | 0 | 2.73 | 2.43 | 83.64 |
| Respiration | 7.68 | 5.12 | 2.33 | 85.97 |
| Fatty Acids and Lipids | 0 | 2.53 | 2.23 | 88.19 |
| Protein Metabolism | 5.48 | 7.56 | 1.78 | 89.97 |
| Cell Wall and Capsule | 9.22 | 8.65 | 1.6 | 91.57 |
| DNA Metabolism | 3 | 4.85 | 1.51 | 93.07 |
| Macromolecular Synthesis | 1.41 | 0 | 1.23 | 94.31 |
| Sulfur Metabolism | 3.16 | 2 | 1.07 | 95.38 |
| Phosphorus Metabolism | 4.24 | 3.05 | 1.06 | 96.44 |
| Cell Division and Cell Cycle | 7.48 | 6.35 | 1.03 | 97.48 |
| Nucleosides and Nucleotides | 2.65 | 3.46 | 0.83 | 98.31 |
| Regulation and Cell signaling | 3.32 | 4.03 | 0.71 | 99.02 |
| Secondary Metabolism | 0 | 0.71 | 0.57 | 99.6 |
| Nitrogen Metabolism | 1.41 | 1.87 | 0.4 | 100 |
